# PARsylated transcription factor EB (TFEB) regulates the expression of a subset of Wnt target genes by forming a complex with β-catenin-TCF/LEF1

**DOI:** 10.1038/s41418-021-00770-7

**Published:** 2021-03-22

**Authors:** Soyoung Kim, Gahyeon Song, Taebok Lee, Minseong Kim, Jeongrae Kim, Hyeryun Kwon, Jiyoung Kim, Wonyoung Jeong, Ukjin Lee, Chaebin Na, Sangwon Kang, Wantae Kim, Je Kyung Seong, Eek-hoon Jho

**Affiliations:** 1 Department of Life Science, University of Seoul, Seoul, Republic of Korea; 2 Confocal Core Facility, Center for Medical Innovation, Seoul National University Hospital, Seoul, Republic of Korea; 3 DKFZ-ZMBH Alliance, Deutsches Krebsforschungszentrum (DKFZ), Heidelberg, Germany; 4 Department of Mathematics, University of Seoul, Seoul, Republic of Korea; 5 Research Center for Cell Homeostasis, Ewha Womans University, Seoul, Republic of Korea; 6 Department of Biochemistry, Chungnam National University, Daejeon, Republic of Korea; 7 Laboratory of Developmental Biology and Genomics, College of Veterinary Medicine, Seoul National University, Seoul, Republic of Korea

**Keywords:** Oncogenes, Autophagy

## Abstract

Wnt signaling is mainly transduced by β-catenin via regulation of the β-catenin destruction complex containing Axin, APC, and GSK3β. Transcription factor EB (TFEB) is a well-known master regulator of autophagy and lysosomal biogenesis processes. TFEB’s nuclear localization and transcriptional activity are also regulated by various upstream signals. In this study, we found that Wnt signaling induces the nuclear localization of TFEB and the expression of Wnt target genes is regulated by TFEB-β-catenin-TCF/LEF1 as well as β-catenin-TCF/LEF1 complexes. Our biochemical data revealed that TFEB is a part of the β-catenin destruction complex, and destabilization of the destruction complex by knockdown of either Axin or APC causes nuclear localization of TFEB. Interestingly, RNA-sequencing analysis revealed that about 27% of Wnt3a-induced genes were TFEB dependent. However, these “TFEB mediated Wnt target genes” were different from TFEB target genes involved in autophagy and lysosomal biogenesis processes. Mechanistically, we found that Tankyrase (TNKS) PARsylates TFEB with Wnt ON signaling, and the nuclear localized PARsylated TFEB forms a complex with β-catenin-TCF/LEF1 to induce the “TFEB mediated Wnt target genes”. Finally, we found that in various types of cancer, the levels of TFEB mediated Wnt target genes exhibit strong correlations with the level of Axin2, which represents the activity of Wnt signaling. Overall, our data suggest that Wnt signaling induces the expression of a subset of genes that are distinct from previously known genes regulated by the β-catenin-TCF/LEF1 complex or TFEB, by forming a transcription factor complex consisting of PARsylated TFEB and β-catenin-TCF/LEF1.

## Introduction

The basic helix-loop helix leucine zipper transcription factor EB (TFEB) is a member of the microphthalmia-associated transcription factor (MITF) subfamily [1]. TFEB recognizes and directly binds to a specific E-box sequence of lysosomal genes and acting as a master regulator of lysosomal function. With several lysosomal genes tending to display coordinated transcriptional behavior, this gene network is termed CLEAR (Coordinated Lysosomal Expression and Regulation) and the E-box sequence at the promoter region of CLEAR genes is regarded as a CLEAR element. Genomic analyses have also identified that TFEB directs target genes involved in the regulation of additional lysosome-associated processes, such as for autophagy, phagocytosis and various immune responses [2–4].

The transcriptional activity of TFEB responds to environmental cues and is regulated through its post-translational modification such as phosphorylation [5]. Under nutrient rich conditions, phosphorylated TFEB is mainly located in the cytoplasm, whereas under starvation or stress conditions, de-phosphorylation of TFEB allows it to enter the nucleus and activate lysosomal gene expression [6]. Multiple kinases that phosphorylate TFEB have been identified; these include mTORC1 [7], ERK2 [6], protein kinase C (PKC), and glycogen synthase kinase 3 (GSK3) [8].

Wnt/β-catenin mediated gene expression is controlled by the transcription co-activator β-catenin. In the absence of Wnt, cytoplasmic β-catenin is located to the Axin complex, which includes Axin, APC (adenomatous polyposis coil), CK1 (casein kinase1) and GSK3. With β-catenin becoming phosphorylated by GSK3β and CK1α in the destruction complex, it is recognized and ubiquitinated by β-TrCP E3 ligase for subsequent proteasomal degradation. Binding of the Wnt ligand to Frizzled and the co-receptor LRP5/6 initiates the Wnt downstream signaling; this leads to relocation of the destruction complex to the plasma membrane via adapter DVL and breaking of the destruction cycle, followed by stabilization of cytoplasmic β-catenin. The accumulated β-catenin then enters the cell nucleus and interacts with TCF/LEF1, resulting in activation of various target genes [9, 10]. GSK3β also phosphorylates numerous substrates besides β-catenin and recent research suggests that inhibition of GSK3β by Wnt signaling also stabilizes multiple proteins including c-MYC with the resultant control of biological processes such as cell division by Wnt [10–13].

Here, we showed that TFEB, a master regulator of expression of genes related to lysosomal biogenesis, acts as a novel mediator of Wnt signaling cascade. We found that Wnt signaling induced PARsylation of TFEB and dissociation of TFEB from the destruction complex, followed by translocation of TFEB to the nucleus. Interestingly, nuclear TFEB induced by Wnt signaling did not induce lysosomal gene expression. RNA-sequencing analysis of cells with TFEB knockdown, which were then Wnt treated, revealed that about 27% of increased genes by Wnt treatment were TFEB dependent; we call these “TFEB-mediated Wnt target” genes. Interestingly, neither ectopic expression of TFEB nor nuclear TFEB induced by starvation induced expression of the TFEB-mediated Wnt target genes, which suggests that nuclear-localized TFEB induced by Wnt is different from the nuclear TFEB induced by starvation. We also found that TNKS1, a poly ADP-ribosylating enzyme, PARsylates TFEB upon Wnt treatment in turn induced nuclear localization TFEB and expression of TFEB-mediated Wnt target genes. The PARsylated TFEB was bound to TCF/LEF1-β-catenin, thus explaining the different profile of TFEB-mediated Wnt target genes from the profile of genes induced by TCF/LEF1-β-catenin or the starvation mediated TFEB.

## Result

### Wnt/β-catenin signaling induces nuclear localization of TFEB

It is known that Wnt/β-catenin signaling represses GSK3β activity and inhibition of GSK3β induces nuclear localization of TFEB [8]. Based on this commonality, we speculated that the nuclear localization of TFEB and expression of genes involved in lysosomal biogenesis for autophagy may be regulated by Wnt signaling. To explore if TFEB subcellular localization and activity are regulated by Wnt signaling, we generated HeLa cells which stably expressed TFEB-EGFP. We found that, consistent with previous studies, nutrient starvation or treatment of GSK3β inhibitor induces nuclear localization of TFEB (Supplementary Fig. S1a, b).

**Fig. 1:**
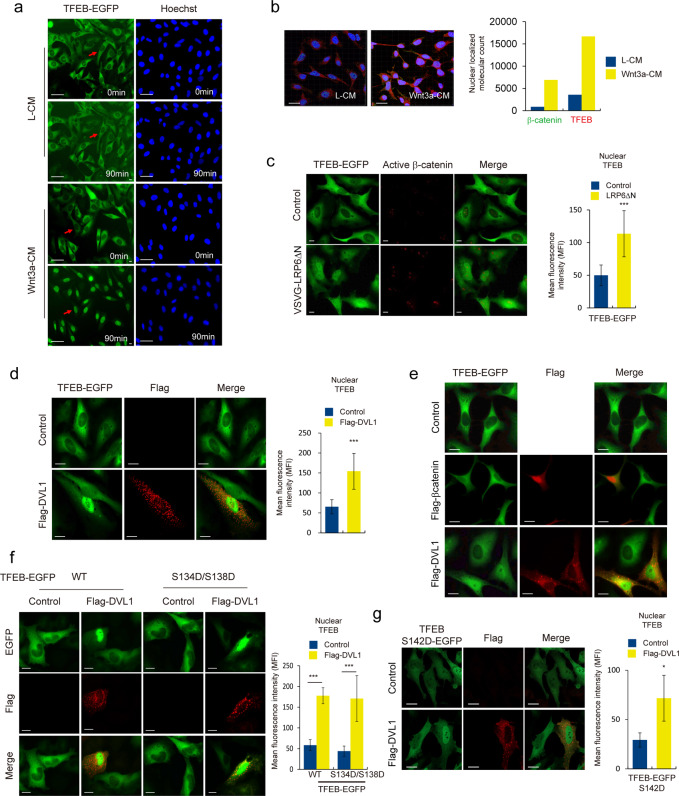
Wnt signaling induces translocation of TFEB into the nucleus. **a** TFEB-EGFP was translocated into nucleus with Wnt3a stimulation. TFEB-EGFP stable cell lines were treated with L-CM or Wnt3a-CM. Cell fluorescence live images were then captured using the live-cell imaging microscope. Nuclei were stained by Hoechst. Scale bar, 50 μm. **b** Levels of endogenous TFEB and β-catenin were increased in the nucleus upon treatment with Wnt3a-CM. HeLa cells were subjected to treatment with L-CM or Wnt3a-CM for 4 h. Active β-catenin and TFEB were labeled with Alexa488-Green and Alexa546-Red, respectively. Images were analyzed with IMARIS image analysis software V.7.6.2 for molecular count of green-colored active β-catenin and red-colored TFEB. Scale bar, 10 μm. **c** Overexpression of LRP6ΔN induced nuclear localization of TFEB-EGFP. The TFEB-EGFP stable cells were transfected with VSVG- LRP6ΔN and subjected to immunofluorescence (IF) analysis. Quantification of nuclear TFEB was shown in right panel. Scale bar, 20 μm. **d** Overexpression of DVL in TFEB-EGFP stable cells induced nuclear localization of TFEB-EGFP. The TFEB-EGFP stable cells were transfected with Flag-DVL1 and subjected to immunofluorescence (IF) analysis. Quantification of nuclear TFEB was shown in right panel. Scale bar, 20 μm. **e** Overexpression of β-catenin had no effect on TFEB localization. Scale bar, 20 μm. **f**, **g** Co-overexpression of DVL and TFEB S134D/S138D-EGFP (**f**) or TFEB S142D-EGFP (**g**) into HeLa cells induced nuclear localization of TFEB mutant forms, respectively. Quantification of nuclear TFEB was shown in right panel. Scale bar, 20 μm. Data information: In (**c**, **d**, **f**, **g**), quantification of mean fluorescence intensity (MFI) of TFEB in the nuclear ROI region from 8 bit confocal images (maximum gray value, 256) is shown. Data are presented as mean ± SEM. *P < 0.05, **P < 0.01, and ***P < 0.005 (Student’s *t* test).

Using live-cell imaging fluorescent microscopy, we found that treatment with Wnt3a-conditioned media (Wnt3a-CM) induced nuclear localization of TFEB-EGFP within 90 min (Fig. 1a); for the same time period, in control conditioned media (L-CM) treated cells, TFEB-EGFP remained in the cytoplasm. Consistently, nuclear accumulation of endogenous TFEB was also observed with Wnt3a-CM treatment of the cells (Fig. 1b and Supplementary Fig. S1c). Besides using Wnt3a-CM, treatment of cells with recombinant Wnt3a protein (rWnt3a) also induced the nuclear translocation of TFEB (Supplementary Fig. S1d). Knockdown of LRP6, a Wnt co-receptor, blocked the increase in nuclear TFEB levels induced by treatment with Wnt3a conditioned media (Supplementary Fig. S1e). Collectively, these data suggested that Wnt signaling induces nuclear localization of TFEB.

To further dissect the level of Wnt signaling pathway responsible for nuclear localization of TFEB, we utilized VSVG-LRP6ΔN (lacking N-terminal extracellular domain) and FLAG-DVL1 constructs that can bypass the interaction between of Wnt ligand and receptor and directly activate the downstream pathway of the receptor complex [14, 15]. Overexpression of either LRP6ΔN or DVL1 constructs promoted nuclear localization of TFEB (Fig. 1c, d and Supplementary Fig. S1f). However, interestingly, overexpression of β-catenin had no effect on localization of TFEB (Fig. 1e). These data suggest that activation of Wnt signaling induces nuclear localization of TFEB and this event occurs upstream of β-catenin stabilization.

### Induced nuclear localization of TFEB upon Wnt signaling activation appears to be independent of the phosphorylation status of TFEB

As subcellular localization of TFEB are strictly regulated by its phosphorylation of specific serine residue [16], we tested whether Wnt signaling or cell starvation promotes de-phosphorylation of TFEB. A faster migration of the TFEB band in the electrophoresis gel was observed in samples treated with various GSK3β inhibitors such as CHIR99021, LiCl and BIO, but not for Wnt3a-CM treatment (Supplementary Fig. S1g). Previously, the interaction between TFEB and 14-3-3 was shown to be reduced in a starvation state of the cell via inhibition of TFEB phosphorylation [7, 17, 18]. Consistent with these previous studies, nutrient withdrawal resulted in reduction of the interaction between TFEB and 14-3-3, whereas this interaction was not reduced by Wnt3a-CM treatment (Supplementary Fig. S1h).

We mutated specific serine residues of TFEB, previously reported to modulate the localization of TFEB, and tested whether the nuclear localization of the mutated TFEB by Wnt treatment would be affected [6, 18]. The phosphomimetic mutants of TFEB (S134D/S138D) and TFEB (S142D) were mainly localized to the cytoplasm. However, ectopic expression of DVL1 could still induce nuclear localization of these mutants (Fig. 1f, g). To further confirm that phosphorylation of S134, S138, and S142 is involved in the response to glucose starvation while Wnt-mediated nuclear localization of TFEB is not controlled by the phosphorylation status of these sites, a phosphomimetic mutant TFEB-EGFP form, in which all Serine 134, 138, and 142 residues were mutated to Aspartate, was tested. Wnt3a-CM treatment was still able to significantly enhance the nuclear levels of this mutant, while glucose starvation had no effect (Supplementary Fig. S1i). These findings suggest that Wnt signaling mediated-nuclear localization of TFEB is regulated via a different mechanism, and is independent of its phosphorylation status that is controlled by starvation.

### Wnt signaling mediated nuclear localization of TFEB is regulated within the β-catenin destruction complex

**Fig. 2:**
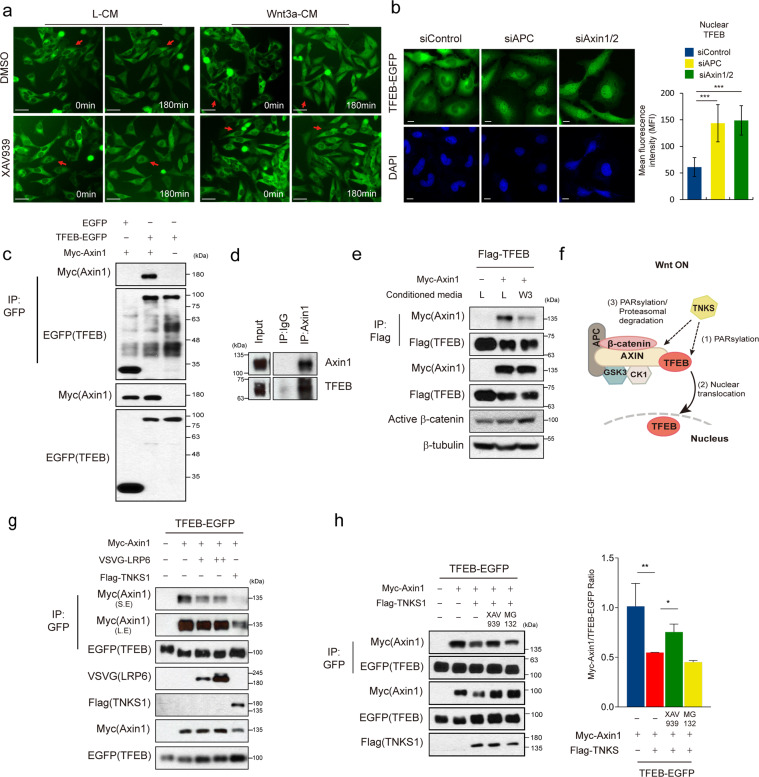
Wnt signaling dependent nuclear localization of TFEB is regulated in the β-catenin destruction complex. **a** Treatment of XAV939 inhibits Wnt3a-mediated nuclear localization of TFEB. TFEB-EGFP stable cells were treated with or without 2 μM XAV939 in L-CM or Wnt3a-CM. Scale bar, 50 μm. **b** Knockdown of the destruction complex component induced nuclear localization of TFEB-EGFP. Immunofluorescence analysis was performed in TFEB-EGFP stably expressing cells transfected with control, Axin1/2 or APC siRNA. Quantification of mean fluorescence intensity (MFI) of TFEB in the nuclear ROI region from 8 bit confocal images (maximum gray value, 256) is shown in right panel. Scale bar, 20 μm. **c** TFEB interacts with Axin1 which is a component of the β-catenin destruction complex. EGFP-TFEB and Myc-Axin1 were co transfected into HEK293T cells. Cell lysates were then immunoprecipitated with anti-GFP antibody and immunoblotted with the indicated antibodies. **d** Axin interacts with TFEB at the endogenous level. HeLa cell lysates were immunoprecipitated with endogenous anti-Axin1 antibody and immunoblotted with endogenous anti-TFEB antibody. **e**, **g**, and **h** Activation of Wnt signaling reduced interaction between TFEB and Axin1. **e** Flag-TFEB and Myc-Axin1-expressing HEK293T cells were treated with Wnt3a-CM, followed by immunoprecipitation with anti-Flag antibody and immunoblotting with the indicated antibodies. **f** Schematic diagram for the prediction of effects of TNKS1 overexpression on dissociation of TFEB from the β-catenin destruction complex. **g** TFEB-EGFP and Myc-Axin1-expressing HEK293T cells were co-transfected with VSVG-LRP6 or Flag-TNKS1. Lysates were immunoprecipitated with anti-GFP antibody and immunoblotted with the indicated antibodies. **h** Treatment of XAV939, but not MG132, blocked TNKS1 dependent reduction of interaction between TFEB and Axin1. TFEB-EGFP and Myc-Axin1 and Flag- TNKS1 expressing HEK293T cells were treated with XAV939 or MG132. Cell lysates were immunoprecipitated with anti-GFP antibody and immunoblotted with the indicated antibodies. Quantification of the ratio of immunoprecipitated Myc-Axin1/TFEB-EGFP of three independent immunoblots was shown in the right panel. Data information: In (**b**) and (**h**), data are presented as mean ± SEM. *P < 0.05, **P < 0.01, and ***P < 0.005 (Student’s *t* test).

As Wnt signaling mediated nuclear localization of TFEB was occurring independently of TFEB’s phosphorylation status and was regulated upstream of β-catenin (Fig. 1f, g and Supplementary Fig. S1i), we examined the possibility whether this TFEB is in the β-catenin destruction complex. It is known that Wnt signaling activation reduces the level of Axin, a concentration-limiting scaffolding protein, leading to increase of β-catenin levels [19]. Tankyrase (TNKS) mediated poly ADP-ribosylation (PARsylation) of Axin is known to induce ubiquitin-dependent degradation of Axin [20, 21]. To examine whether Wnt induces the nuclear localization of TFEB through dissociation of TFEB from the β-catenin destruction complex via Axin degradation, the Axin degradation in Wnt3a-CM treated cells was blocked by using IWR-1 or XAV939, small molecules that inhibit TNKS [22, 23]. Treatment of cells with IWR-1 endo or XAV939 blocked Wnt3a-CM mediated nuclear localization of TFEB, whereas treatments with IWR-1 exo or DMSO, the negative controls for IWR-1 endo and XAV939, respectively, did not block this nuclear localization (Fig. 2a and Supplementary Fig. S2a). Furthermore, knockdown of Axin1/2 or APC, which is core component of destruction complex, induced nuclear localization of TFEB (Fig. 2b, Supplementary Fig. S2b). These results indicate that translocation of TFEB may be regulated by the β-catenin destruction complex.

To prove whether TFEB is sequestered in the cytoplasmic β-catenin destruction complex, we examined the interaction between TFEB and Axin. Immunoprecipitation (IP) assays revealed that TFEB and Axin interacts at the overexpression condition as well as endogenous level (Fig. 2c, d). Interestingly, we found that TFEB dissociates from Axin1 either upon Wnt3a-CM treatment of the cells or with LRP6 overexpression (Fig. 2e and Supplementary Fig. S2c). These data suggest that TFEB is part of the β-catenin destruction complex and activation of Wnt signaling releases TFEB from the destruction complex, allowing nuclear localization of TFEB to proceed (step (2) in Fig. 2f). Furthermore, overexpression of TNKS1, which promotes degradation of Axin, also promoted dissociation of TFEB from Axin (Fig. 2g), suggesting two possibilities for the release of TFEB from β-catenin destruction complex: (i) it occurs due to degradation of Axin (step (3) in Fig. 2f) or (ii) via PARsylation of TFEB (step (1) in Fig. 2f). To test these possibilities, we inhibited proteasomal degradation of Axin with MG132 or blocked TNKS activity through treatment with XAV939. Intriguingly, the release of TFEB under TNKS1 overexpression was inhibited by treatment with XAV939 but not MG132 (Fig. 2h). Therefore, TNKS1 dependent PARsylation of TFEB but not Axin degradation might be responsible for release of TFEB from the β-catenin destruction complex.

### TNKS1 dependent PARsylation of TFEB is necessary for Wnt signaling mediated nuclear localization of TFEB

**Fig. 3:**
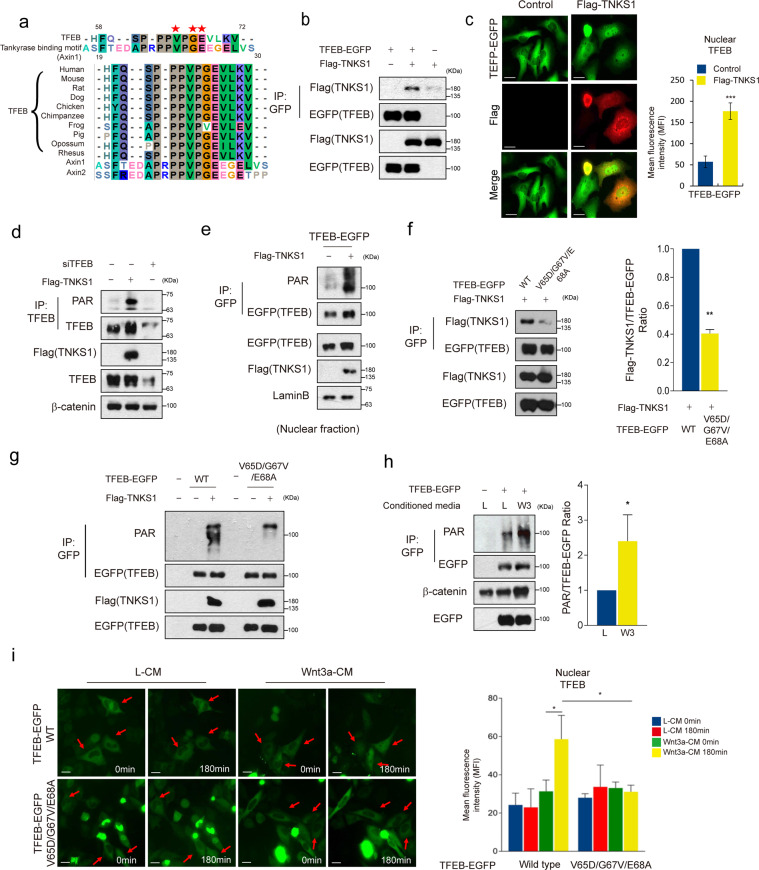
Wnt signaling regulates TNKS1 mediated PARsylation of TFEB, followed by dissociation from destruction complex to nuclear localization. **a** The TNKS1 binding motif of TFEB is evolutionarily conserved. Colored are the highly conserved sequences. **b** TFEB interacted with TNKS1. TFEB-EGFP and Flag-TNKS1 were co-transfected into HEK293T cells. Cell lysates were immunoprecipitated with anti-GFP antibody and immunoblotted with the indicated antibodies. **c** Overexpression of TNKS1 induced nuclear localization of TFEB. TFEB-EGFP stable cells were transfected with the corresponding plasmid as indicated and were the subjected to IF analysis. Quantification of mean fluorescence intensity (MFI) of TFEB in the nuclear ROI region from 8 bit confocal images (maximum gray value, 256) was shown in the right panel. Scale bar, 20 μm. **d** Overexpression of TNKS1 induced PARsylation of TFEB. Flag-TNKS1 was transfected into HeLa cells. Cells were lysed with RIPA buffer containing the poly(ADP-ribose) glycohydrolase inhibitor, ADP-HPD (5 μM). Lysates were then immunoprecipitated with anti-TFEB antibody and immunoblotted with Poly(ADP-Ribose)Polymer antibody. **e** Nuclear TFEB was highly PARsylated by overexpression of TNKS1. HEK293T cells were transfected with the indicated plasmids and nuclear lysates were used in immunoprecipitations. Nuclear lysates and immunoprecipitates were immunoblotted with the indicated antibodies. **f** Val-65, Gly-67 and Glu-68 of TFEB are required for interaction with TNKS1. Flag-TNKS1 with TFEB-EGFP or TFEB-EGFP mutant were transfected into HEK293T cells and cell lysates were immunoprecipitated with anti-GFP antibody and immunoblotted with the indicated antibodies. Quantification of the ratio of immunoprecipitated Flag-TNKS1/TFEB-EGFP of three independent immunoblots was shown in the right panel. **g** Mutant form of TFEB, which has a lower affinity for TNKS1 than the wild type TFEB, showed a reduced level of PARsylation under the TNKS1 overexpression condition. HEK293T cells were transfected with the indicated plasmids and cell lysates were immunoprecipitated with anti-GFP antibody and immunoblotted with the indicated antibodies. **h** Treatment with Wnt3a-CM increased PARsylation of TFEB. TFEB-EGFP expressing HEK293T cells were treated with Wnt3a-CM for 4 h. Cells were lysed with RIPA buffer containing poly (ADP-ribose) glycohydrolase inhibitor, ADP-HPD (5 μM). Lysates were immunoprecipitated with anti-GFP antibody and immunoblotted with PARsylation (PAR) specific antibody. Quantification of the ratio of immunoprecipitated PARsylated TFEB/TFEB-EGFP of three independent immunoblots was shown in the right panel. **i** Treatment with Wnt3a-CM induced nuclear localization of wild type TFEB, but not TNKS-binding deficient TFEB mutant. HeLa cells transfected with TFEB-EGFP or TFEB-EGFP-DVA plasmid were treated with L-CM or Wnt3a-CM. Quantification of mean fluorescence intensity (MFI) of TFEB in the nuclear ROI region from 8 bit confocal images (maximum gray value, 256) was shown in right panel. Scale bar, 20 μm.

Previous reports have shown that binding partners or substrates of TNKS possess a consensus sequence known as the TNKS binding motif (TBM: RXXPDG) [24, 25]. Mutagenesis analysis revealed that N-terminal domain of Axin is necessary for interaction with TNKS [26]. Interestingly, TFEB has a TNKS binding residue, which is also present in Axin (Fig. 3a). TFEB interacts with TNKS1 and overexpression of TNKS1 induces nuclear localization of TFEB (Fig. 3b, c and Supplementary Fig. S3a). Within catalytic domain of TNKS1, three Zinc-coordinating Cys residues (C1234, C1242, C1245) are necessary for poly ADP-ribose polymerase (PARP) activity of TNKS [27] (Supplementary Fig. S3b). To examine whether TNKS required its PARP activity for inducing nuclear localization of TFEB, we measured the levels of TFEB in the nucleus after ectopic expression of TNKS1 mutants. Wild type TNKS1, but not TNKS1 mutants (C1234S and C1242S), increases nuclear TFEB levels (Supplementary Fig. S3c). These results indicate that PARP activity of TNKS1 is necessary for the nuclear localization of TFEB. We then tested whether TFEB is PARsylated by TNKS1. Overexpression of TNKS1 induced PARsylation of endogenous TFEB and TFEB-EGFP (Fig. 3d and Supplementary Fig. S3d). Furthermore, we found that nuclear TFEB is highly PARsylated (Fig. 3e). We performed a PARsylation assay using deletion constructs of TFEB and found that TFEB WT and 1-292 fragments, but not the 293–476 fragment of TFEB is PARsylated (Supplementary Fig. S3e).

We tested whether TFEB and TNKS1 can actually interact and whether this interaction would be essential for the induced nuclear localization of TFEB by TNKS1. Mutant TFEB (TFEB V65D/G67V/E68A, herein referred to as TFEB DVA) exhibited a much reduced interaction with TNKS1 and PARsylation levels compared to wild type TFEB (Fig. 3f, g). Immunoblotting of the nuclear fractions showed that the nuclear levels of TFEB DVA are not increased after overexpression of TNKS1 (Supplementary Fig. S3f). Interestingly, interaction between wild type TFEB, but not TFEB DVA, and Axin was severely reduced with the overexpression of TNKS1 (Supplementary Fig. S3g and Fig. 2g).

Treatment of cells with Wnt3a-CM further increased PARsylation of TFEB (Fig. 3h). Surprisingly, unlike wild type TFEB translocating to the nucleus following treatment with Wnt3a-CM, TFEB DVA did not translocate to the nucleus under treatment with Wnt3a-CM (Fig. 3i). Consistently, the level of wild type TFEB, but not the TFEB DVA mutant, in the nuclear fraction was increased upon treatment with Wnt3a-CM (Supplementary Fig. S3h). These results suggest that TNKS-dependent PARsylation of TFEB is necessary for Wnt signaling-mediated nuclear localization of TFEB.

After reaching the conclusion described above, we next wondered how knockdown of APC or Axin1/2 alone, which may not induce PARsylation of TFEB, can induce nuclear localization of TFEB (Fig. 2b, Supplementary Fig. S2b). We hypothesized that knockdown of Axin1/2 or APC induces autocrine Wnt signaling. We found that knockdown of Axin1/2 or APC increased TFEB PARsylation, even in the absence of Wnt treatment, and knockdown of TNKS1 reduced APC knockdown-mediated induction of TFEB nuclear localization (Supplementary Fig. S4a–c). Real time PCR analysis showed increased expression of the canonical Wnt ligands Wnt2 and Wnt3a, but not the non-canonical Wnt ligand Wnt5a (Supplementary Fig. S4d). Overall, our data suggest that knockdown of either Axin1/2 or APC increases autocrine Wnt signaling and thereby enhances TNKS1 activity on TFEB, ultimately leading to nuclear localization of TFEB.

### Wnt signaling dependent nuclear-localized TFEB does not induce expression of genes involved in lysosomal biogenesis

**Fig. 4:**
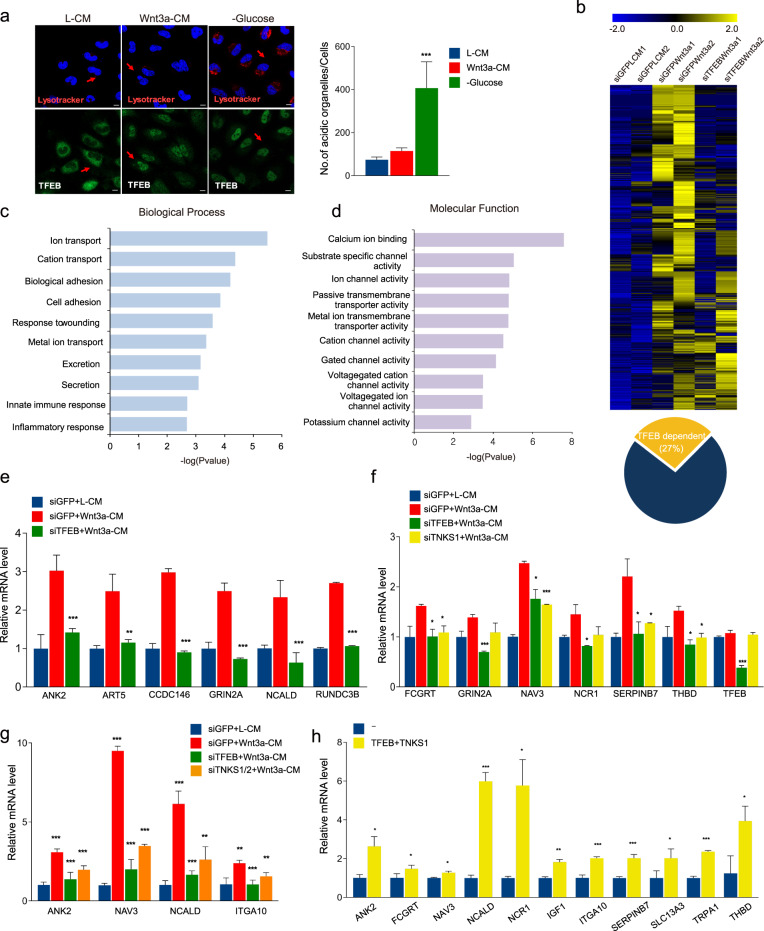
Wnt signaling increases expression of target genes through TFEB. **a** Wnt3a-mediated nuclear-localized TFEB does not induce lysosomal biogenesis. TFEB-EFGP stable cells were treated with the indicated media for 4 h. Acidic organelles were visualized by treatment with the LysoTracker dye. Both Wnt3a-CM treatment and glucose starvation induced nuclear localization of TFEB-EGFP (Bottom). Only glucose starvation, but not Wnt3a-CM treatment, induced the increased number of lysosomes (Top). Quantification of number of acidic organelles/cells was shown in the right panel. Scale bar, 10 μm. **b** Heat map analysis of expression profiles after treatment with Wnt3a-CM under siGFP or siTFEB conditions. About 27% genes among the genes upregulated by Wnt3a-CM treatment was TFEB-dependent. **c**, **d** Top 10 GO analysis of “TFEB-mediated Wnt target genes”. Bar chart shows classification of Biological Processes (**c**) or Molecular Function (**d**). Bars represent the *p* value for the specified category. **e** Confirmation of “TFEB-mediated Wnt target genes”. siGFP or siTFEB transfected HeLa cells were treated with L-CM or Wnt3a-CM for 6 h and qPCR was performed to measure expression of randomly selected genes among “TFEB-mediated Wnt target genes”. **f**, **g** Wnt signaling did not increase the expression of “TFEB-mediated Wnt target genes” upon TNKS1 (**f**) or TNKS1/2 (**g**) knockdown as well as TFEB knockdown. siGFP, siTFEB, siTNKS1, or siTNKS1/2 transfected HeLa cells were treated with L-CM or Wnt3a-CM. qPCR was performed to measure expression of “TFEB-mediated Wnt target genes”. **h** Co-transfection TFEB and TNKS1 increased expression of “TFEB-mediated Wnt target genes”. qPCR was performed for “TFEB-mediated Wnt target genes” in TFEB-EGFP and Flag-TNKS1 expressing HeLa cells. Data information: In (**e–g**), data are presented as mean ± SEM. *P < 0.05, **P < 0.01, and ***P < 0.005 (Student’s *t* test).

Since TFEB is a master regulator of genes involved in lysosomal biogenesis, we examined whether Wnt signaling mediated nuclear-localized TFEB enhances lysosomal activity. Lysotracker dye is a specific marker of acidic organelles such as lysosomes. Either treatment with Wnt3a-CM or glucose starvation of the cells induces nuclear localization of TFEB (Fig. 4a bottom); however, only glucose starvation, and not Wnt3a-CM treatment, increased the extent of acidic organelle compartments (Fig. 4a top). For additional confirmation, we also examined functional lysosome levels by using DQ-BSA. Fluorescence of DQ-BSA is strongly quenched, but in the starvation condition, traffics to lysosomes and is cleaved to a smaller fragment and results in emitting a green fluorescent signal [28]. Although both rWnt3a and glucose starvation induced nuclear localization of TFEB (Supplementary Fig. S5a bottom), only glucose starvation increased the number of functional lysosomes (Supplementary Fig. S5a top). These data suggest that Wnt3a-mediated nuclear-localized TFEB does not induce lysosomal biogenesis. Real time PCR analysis also showed that glucose starvation or overexpression of TFEB, but not treatment with Wnt3a-CM, increases TFEB-dependent lysosomal target gene expression (Supplementary Fig. S5b, c). From these results, we conclude that the nuclear TFEB induced by Wnt3a might have a different role than activating the expression of genes related to lysosomal biogenesis.

### A subset of genes induced by Wnt signaling are dependent on nuclear-localized TFEB

Our RNA sequencing analysis showed that around 27% of the up-regulated genes with Wnt3a-CM treatment are dependent on TFEB (Fig. 4b). A GO analysis of these TFEB mediated Wnt-dependent targets showed that the prominent biological processes involved in this pathway were linked to ion transport and the inflammatory response (Fig. 4c). For specific GO molecular functions, ion binding and channel activity were identified (Fig. 4d). Real time PCR analysis showed that a knockdown of TFEB inhibited expression of “TFEB-mediated Wnt target” group of genes (Fig. 4e, for Wnt3aCM treatment; and Supplementary Fig. S5d, for rWnt3a treatment). Knockdown of TFEB has no effect on nuclear localization of β-catenin, indicating that blocking expression of “TFEB-mediated Wnt target genes” by TFEB knockdown was not due to an indirect inhibition of nuclear localization of β-catenin (Supplementary Fig. S5e). We also found that expression of “TFEB-mediated Wnt target genes” is not regulated by glucose starvation (Supplementary Fig. S5f). Collectively, nuclear-localized TFEB with Wnt activation induces expression of a set of genes distinct from those involved in lysosomal biogenesis.

We tested whether TNKS is necessary for expression of “TFEB mediated Wnt target genes”. Real time PCR analysis revealed that knockdown of TNKS1, or both TNKS1 and 2, abrogated the induced expression of “TFEB mediated Wnt target genes” (Fig. 4f, g). Conversely, co-overexpression of TFEB and TNKS1 increased the expression of these genes (Fig. 4h). These data suggest that expression of “TFEB mediated Wnt target genes” is dependent on TNKS-dependent PARsylation of TFEB.

### TFEB forms a trimeric complex with β-catenin-TCF/LEF1 to induce the expression of TFEB-mediated Wnt target genes

**Fig. 5:**
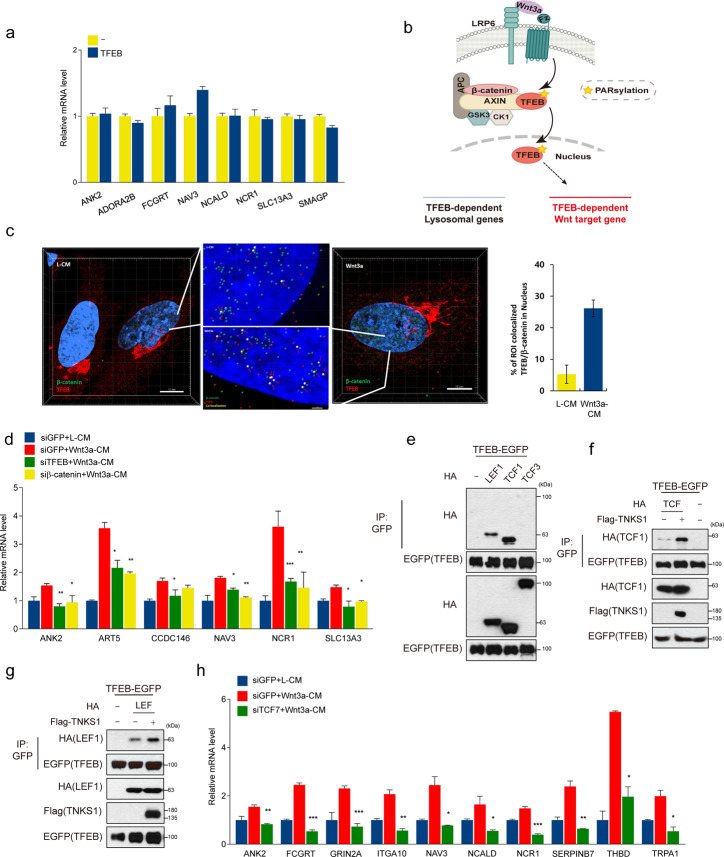
Expression of “TFEB-mediated Wnt target genes” is β-catenin and TCF/LEF dependent. **a** TFEB alone did not induce TFEB mediated Wnt target gene expression. qPCR was performed for “TFEB-mediated Wnt target genes” in HeLa cells transfected with control or TFEB-EGFP plasmids. **b** A schematic diagram of the proposed model. **c** Colocalization spot analysis. β-catenin and TFEB levels in HeLa cells were detected with Alexa 488 and Alexa 568, respectively. Estimated XY diameter of each sphere is 0.25 µm and z diameter is 0.5 µm, and PSF was elongated along the z-axis using Imaris 9.2 image analysis software (Bitplane). Scale bar, 10 μm. **d** Wnt signaling did not increase TFEB mediated Wnt target gene expression when β-catenin was knockdowned as well as for TFEB knockdown. siGFP or siTFEB or siβ-catenin transfected HeLa cells were treated with L-CM or Wnt3a-CM for 6 h. qPCR was performed to measure expression of TFEB-mediated Wnt target genes. **e** TFEB interacted with TCF1 and LEF1. HEK293T cells were transfected with the indicated plasmids. After 24 h, cell lysates were immunoprecipitated with anti-GFP antibody and analyzed by Western blotting. **f**, **g **Overexpression of TNKS1 enhanced interaction between TFEB and TCF1 (**f**) or LEF1 (**g**), respectively. HEK293T cells were transfected with the indicated plasmids. After 24 h, cell lysates were immunoprecipitated with anti-GFP antibody and analyzed by Western blotting. **h** Wnt signaling did not induce expression of “TFEB-mediated Wnt target genes” under the TCF1 knockdown. siGFP or siTCF7 (the gene that encodes TCF1 in humans) transfected HeLa cells were treated with L-CM or Wnt3a-CM for 6 h. qPCR was performed to measure expression of TFEB-mediated Wnt target genes. Data information: In (**h**), data are presented as mean ± SEM. *P < 0.05, **P < 0.01 and ***P < 0.005 (Student’s *t* test).

Overexpression of TFEB only had minimal effects on the expression of TFEB-mediated Wnt target genes (Fig. 5a). These data raised the question on how nuclear-localized TFEB by Wnt treatment can selectively regulate the expression of TFEB-mediated Wnt target genes (Fig. 5b). Since Wnt signaling induces nuclear localization of β-catenin, we hypothesized that β-catenin together with TFEB might be needed for the expression of the TFEB-mediated Wnt target genes. Confocal microscopy analysis interestingly revealed that TFEB and β-catenin are strongly co-localized in the nucleus after Wnt3a-CM treatment of the cells (Fig. 5c and Supplementary Fig. S6a). Clear reduction of signal on Western blot and immunofluorescence analysis showed the specificity of TFEB antibody (Supplementary Fig. S6b, c). Consistently, either TFEB knockdown or β-catenin knockdown reduced the expression of “TFEB-mediated Wnt target genes” (Fig. 5d). However, overexpression of TFEB and β-catenin could not induce expression of these genes in the absence of Wnt ligand stimulation (Supplementary Fig. S6d). Collectively, our data suggest that both TFEB and β-catenin are necessary but not sufficient for expression of “TFEB-mediated Wnt target genes”. It may be possible that PARsylated TFEB upon activation of Wnt signaling is responsible for induction of these target genes.

Previous reports suggested that MITF, a member of MiT-family of transcription factors similar to TFEB [29]. Since TFEB and MITF have sequence homology [5], we tested whether PARsylated TFEB may also form a complex with TCF/LEF1. We found that TFEB interacts with both TCF1 and LEF1, but not TCF3 (Fig. 5e). High resolution confocal microscopy analysis showed that Wnt3a-CM treatment enhanced the interactions between β-catenin and LEF1, and between LEF1 and TFEB (Supplementary Fig. S6e). The percentage of β-catenin/TFEB/LEF triple co-localization spots (Yellow color) in nuclei was significantly increased upon treatment with Wnt3a-CM (Supplementary Fig. S6f). Moreover, the interaction between TFEB and TCF1 or LEF1 was increased by overexpression of TNKS1 (Fig. 5f, g). Consistently, co-expression of TFEB and TCF1 with TNKS1 significantly induced the expression of “TFEB-mediated Wnt target genes” (Supplementary Fig. S6g). Consistently, knockdown of TCF-1 inhibited Wnt3a-CM-induced TFEB-mediated Wnt target gene expression (Fig. 5h). Taken together, Wnt-mediated TNKS-dependent PARsylation of TFEB forms a complex with TCF/LEF1 to regulate the expression of TFEB-mediated Wnt target genes.

### Potential involvement of TFEB and TFEB-mediated Wnt target genes in cancer progression

**Fig. 6:**
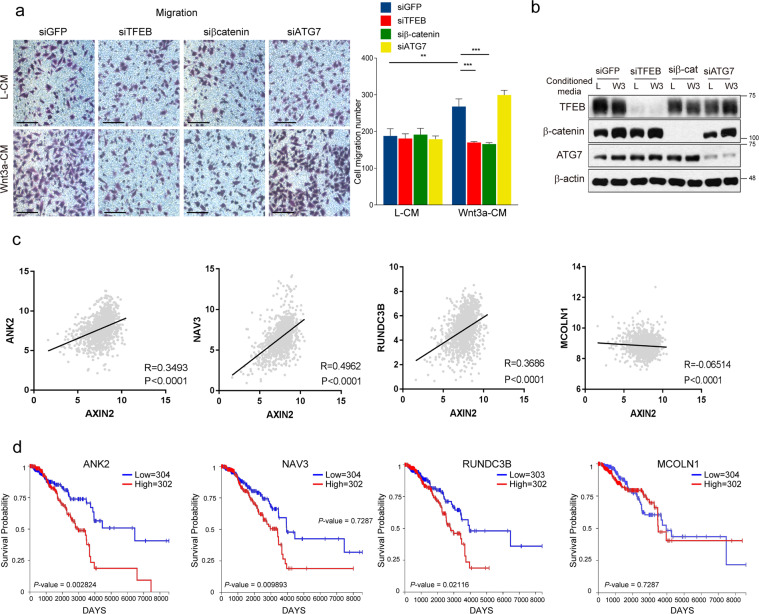
Knockdown of TFEB abolished Wnt3a-dependent cell migration and matrigel invasion enhancement. **a** siRNA targeting TFEB or β-catenin but not Atg7 transfected HeLa cells exhibited impaired cancer cell migration compared with Wnt3a-CM treated cells. Quantification of cell migration ability was shown in the right panel. The statistical analyses represent average values of 3 different areas from a representative experiment. Scale bar, 100 μm. **b** Western blot analysis revealed that the protein expression of TFEB, β-catenin or ATG7 was reduced after the transfection with each siRNA. **c** TCGA gene expression data set of breast cancer patients show positive correlation between Axin2 and TFEB-mediated Wnt target gene (ANK2, NAV3, RUNDC3B) but not TFEB-mediated lysosomal gene (MCOLN1). The Correlation coefficients (*r*) in this figure were calculated by Pearman’s linear correlation. Two-tailed *P* value was used for analyzing statistical significance **d** Kaplan–Meier plot shows that high expression of TFEB-mediated Wnt target genes but not lysosomal gene is associated with poor prognosis of breast cancer patients.

The activation of Wnt signaling promotes cell migration [30]. Consistently, the knockdown of β-catenin significantly impaired HeLa cell migration and matrigel invasion which was enhanced upon the treatment of Wnt3a CM (Fig. 6a–b and Supplementary Fig. S7a, b). Interestingly, both enhanced cell migration and matrigel invasion induced by Wnt3a CM were abrogated by the knockdown of TFEB, but not by the knockdown of Autophagy related 7 (ATG7, an essential regulator of autophagosome assembly) (Fig. 6a, b and Supplementary Fig. S7a, b). Although it is still speculative at this juncture, this data further corroborates our hypothesis that TFEB acts as a mediator of Wnt signaling but not as an autophagy regulator in Wnt activation condition.

To examine whether there is a correlation between the level of Wnt signaling and TFEB mediated Wnt target genes in various types of cancer, we referred to TCGA for breast cancer and bladder cancer or GDC TCGA database for lung squamous cell carcinoma (LUSC). The levels of TFEB mediated Wnt target genes (ANK2, NAV3, FCGRT, IGF1, RUNCD3B) but not TFEB-mediated lysosomal gene (MCOLN1) exhibit strong correlations with the level of Axin2, which represents the activity of Wnt signaling [31] (Fig. 6c and Supplementary Fig. S7c and S7e). Consistently, a high expression level of TFEB-mediated Wnt target genes, but not MCOLN1, was associated with poor prognosis in cancer patients (Fig. 6d and Supplementary Fig. S7d and S7f). To further validate the role of TFEB in cancer progression, we examined nuclear TFEB levels in SW620 (colon cancer cell line) cells and found that they were much higher than in CCD-18Co (normal colon cell line) cells (Supplementary Fig. S8a). Consistently, knockdown of TFEB in HT29 cells (a colorectal cancer cell line that has an APC mutation) resulted in reduced expression of ‘TFEB mediated Wnt target genes’ (Supplementary Fig. S8b). Overall, these data imply that TFEB mediates Wnt signaling for cancer progression.

## Discussion

**Fig. 7:**
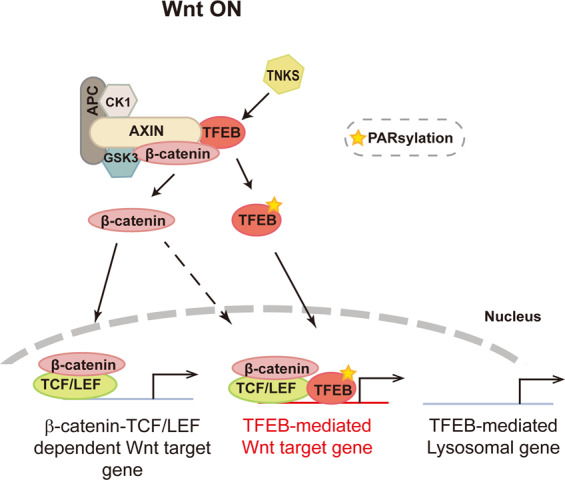
A schematic of the proposed model. Refer to the Discussion for more details.

The findings presented here demonstrate that TFEB, which is known as a master regulator of genes related to lysosomal biogenesis, is a novel terminal regulator of Wnt/β-catenin signaling. Since Wnt signaling suppresses GSK3β [32], known to control nuclear localization of TFEB, we initially postulated that Wnt signaling may regulate autophagy; however, it turned out that nuclear-localized TFEB due to activation of Wnt did not induce expression of the genes involved in lysosomal biogenesis. Interestingly, we found that TFEB is a component of the β-catenin destruction complex. TNKS, activated by Wnt signaling by yet an unknown mechanism, PARsylates TFEB and dissociates TFEB from the β-catenin destruction complex, which then leads to nuclear localization of TFEB in the Wnt ON situation. Nuclear β-catenin and PARsylated TFEB induced by Wnt signaling form a complex with TCF/LEF transcription factors and enhance the expression of “TFEB-mediated Wnt target genes”, rather than the expression of genes related to lysosomal biogenesis (Fig. 7). Overall, our data suggest that the expression of genes induced by Wnt signaling can be modulated by the β-catenin-TCF/LEF-TFEB complex in addition to the β-catenin-TCF/LEF complex, and provides a novel therapeutic point for treatment of Wnt pathway related diseases.

Unlike glucose starvation for which nuclear TFEB transcribes classical CLEAR (Coordinated Lysosomal Expression and Regulation) element containing genes, nuclear TFEB induced by Wnt signaling differentially recognized TFEB target genes independent of CLEAR. Since Wnt signaling induces PARsylation of TFEB, we speculated that PARsylation of TFEB contributes not only to its nuclear localization but also to its genomic occupancy. The latter might be achieved by PAR binding proteins (PAR readers), which would interact with PARsylated TFEB to recruit it to specific genomic locations as many of PAR readers are involved in transcription regulation [33]. PARsylated TFEB may occupy the regulatory regions of “TFEB-mediated Wnt target genes” and bring the β-catenin/TCF complex to initiate transcription.

The relationship between the autophagy process and Wnt signaling is very complex. Several studies have shown that autophagy under starvation conditions reduces Wnt signaling by inducing degradation of Wnt pathway components, DVL [34] and β-catenin [35]. In addition, it is known that β-catenin-TCF4 inhibits the formation of auto-phagosome by reducing the expression of SQSTM1/P62 [35]. Surprisingly, our data suggest that TFEB enters the nucleus when the Wnt signaling is ON (Fig. 1), but it has no effect on the expression of genes related to autophagy (Supplementary Fig. S5c). In addition, the result of cell migration and matrigel invasion assay suggests that TFEB is essential for the Wnt3a mediated up-regulation of these activities not as a regulator of autophagy but as a terminal mediator of Wnt signaling (Fig. 6).

In our model, we suggest that TFEB is a key regulator of various processes, sensitively responding to the surrounding environment of the cell, such as starvation or high Wnt levels, and transmits the appropriate signal. During starvation, TFEB moves to the nucleus and activates auto-lysosomal biogenesis to maintain cellular homeostasis, but in the Wnt ON condition, TFEB enters the nucleus and regulates homeostasis by inducing the expression of Wnt-TFEB target genes such as for mediating ion transport. We have therefore extended the role of TFEB as a master regulator of cellular homeostasis.

Although much effort has been made globally in various laboratories to develop potent modulators of Wnt/β-catenin signaling, only a few such drug candidates have reached clinical trials [36]. Therapies targeting Wnt signaling have been difficult for a safe adaptation as many components regulating Wnt signaling are also involved in various other cellular processes [37] and most clinical studies have focused on target genes that are regulated by β-catenin. Identification of novel targets and determination of their precise mechanisms will lead us to better novel therapeutic applications. As TFEB is a newly described mediator of Wnt signaling, further studies on the regulation and the mechanisms for the expression of “TFEB-mediated target genes” and their biological significance are required, allowing new ways to target human disease caused by dysregulation of Wnt-β-catenin-TFEB signaling.

## Materials and methods

### Cell lines

HEK293T, HeLa, and TFEB-EGFP stable cells were cultured in Dulbecco’s modified Eagle’s Medium (DMEM, Lonza) supplemented with 10% FBS and 1% antibiotics at 37 °C in a humidified 5% CO_2_ incubator. For the glucose-starvation experiment, we mixed no-glucose DMEM (Thermo Fisher Scientific) with 10% FBS and 1% antibiotics.

### Cell transfection

For transient transfection, HEK293T cells were transfected with each plasmid by the calcium phosphate precipitation as described previously [38]. For HeLa cells, plasmids were transfected either with 10 mM polyethylenimine (PEI, Sigma), Turbofect (Thermo Fisher Scientific), or Lipofectamine 2000 (Invitrogen) under the manufacturer instructions.

### Western blotting and antibodies

For Western blotting, cells were suspended in lysis buffer (20 mM Tris-Cl, pH 7.4, 150 mM NaCl, 1% Triton X-100, 1 mM EGTA, 1 mM EDTA, 2.5 mM sodium pyrophosphate, 1 mM β-glycerophosphate, 1 mM Na_3_VO_4_, 1 mM PMSF, and 1 µg/ml leupeptin) for 30 min on ice and centrifuged at 13,000 x rpm for 30 min. Supernatants were collected for subsequent protein assays. For Western blotting, protein samples were separated using 10% SDS-polyacrylamide gel electrophoresis (PAGE) and transferred to a PVDF membrane. The blocked transfer membrane was incubated overnight with primary antibodies at 4 °C. To detect the appropriate proteins, antibodies specific for anti-HA, Lamin B, TNKS1, EGFP (for Western blots, from Santa Cruz and for immunoprecipitation, from Invitrogen), TFEB (Cell Signaling or Bethyl Laboratories), Axin1 (Cell Signaling), β-actin, Flag, VSVG (Sigma), Poly(ADP-Ribose)Polymer (Abcam), Myc(Abm), β-catenin (BD Bioscience), active-β-catenin (Millipore), β-tubulin (Gene Tex) were used.

### Subcellular fractionation

Harvested cells were lysed in Buffer A (10 mM Tris at pH 7.4, 10 mM KCl, 3 mM MgCl_2_, 0.5% NP-40, 100x PMSF) on ice for 20 min and centrifuged at 1500 × *g*, 4 °C for 5 min. Supernatants were collected in a new Eppendorf tube (for the cytosolic fraction). The pellets were washed with 150 μL nuclear washing buffer (10 mM HEPES at pH 7.9, 10 mM KCl, 1.5 mM MgCl_2_, 0.34 M sucrose) and centrifuged at 13,000 rpm, 4 °C for 5 min. After removing these supernatants, the pellets were incubated in Buffer B (20 mM Tris at pH 8.0, 0.42 M NaCl, 0.2 mM EDTA, 10% glycerol, 2 mM DTT, 200x PMSF) with homogenization using a syringe and being incubated on ice for 20 min, followed by centrifugation at 17,000 × *g*, 4 °C for 20 min. These supernatants were collected in a new Eppendorf tube (for the nuclear fraction). This method has been slightly modified from Sung et al, 2019.

For immunoprecipitation from nuclear lysates, Nuclear/Cytosol Fractionation Kit (BioVision) was used.

### Immunoprecipitations

For immunoprecipitation, incubation of 800–1000 µg of lysates was performed with various antibodies including anti-GFP (Invitrogen), anti-TFEB (Cell signaling or Bethyl Laboratories) and IgG control (Stratagene), overnight at 4 °C and followed by incubation with Protein G (Millipore) or Protein A/G Plus agarose beads (Santa Cruz) for 2 hr. Before the bead incubations, the beads were blocked with 5% milk in lysis buffer. After incubation with the lysates, the beads were five-times washed with the lysis buffer; they were then pelleted and boiled in SDS sample buffer. These samples were then subjected to PAGE and Western blotting. For detection of PAR, harvested cells were lysed with RIPA buffer (50 mM Tris pH 8.0, 150 mM NaCl, 0.1% SDS, 1% NP-40, 0.5% sodium deoxycholate, 1 mM PMSF) on ice for 30 min and centrifuged at 13,000 rpm at 4 °C for 10 min. Supernatants were used for immunoprecipitation, where for nuclear lysates, 100 μg of nuclear proteins were incubated with anti-GFP (Invitrogen) antibody in lysis buffer, followed by bead capture and washing as described above.

### Dual luciferase assay

HEK293T cells were seeded in triplicate on 12-well plates and transfected with pSuperTop (0.5 μg), pGC1a (0.5 μg), or pRL-TK (0.05 μg) plasmids. The indicated plasmid was co-transfected into HEK293T or HeLa cells, and 24 h after transfection, the cells were lysed and luciferase activity was measured using a dual-luciferase reporter assay system (Promega) according to the manufacturer’s instructions. Luciferase activity was measured by a GLOMAX 20/20 luminometer (Promega). Transfection efficiency was normalized to the internal control, thymidine kinase promoter-driven Renilla luciferase (pRL-TK).

### HeLa TFEB-EGFP stable cell line

For establishment of the stable cell line, continuously expressing TFEB-EGFP, HeLa cells were transfected with TFEB-EGFP (plasmid #38119, Addgene) by Turbofect transfection reagent (Thermo). On the following day, the transfected HeLa cells were cultured in the selection media containing 400 μg/ml of G418 for 4 days consecutively. The media was replaced every 4 days with an ever-increasing dose of G418 of up to 1,000 μg/ml for 16 days. After this selection period, stably green fluorescence positive colonies were isolated via flow cytometry (FACS). FACS ARIA2 (BD) was used for the sorting with the FACS data analyzed by FACSDiva version 6.1.3 software (BD).

### Immunofluorescence staining and analysis

Cells were seeded in a 38-mm confocal dish (Cat # 100350, SPL) or on glass coverslips in 6-well plates. For the immunofluorescent staining, the cells were fixed for 20 min in 4% paraformaldehyde in PBS and then permeabilized with 0.1% Triton X-100 for 15 min at room temperature. Samples were washed 4 times with PBS. For pre-blocking, the cells were then incubated in 5% bovine serum albumin (BSA) in PBS for 1 h and then overnight incubated with primary antibodies. Primary antibodies were against TFEB (Bethyl Laboratories), β-catenin (BD Bioscience), active- β-catenin (Millipore) and Flag (Sigma). Samples were then washed with 1% BSA in PBS three times and incubated with the secondary antibody conjugated with Alexa488 or Alexa 546 for 1 h. Localization of the proteins was analyzed and imaged with confocal microscopy (see below for details). The Values for Nuclear TFEB were calculated using ImageJ software (Max intensity: 255).

### RNA isolation and real-time PCR

Total RNA was isolated using the TRIzol reagent (Sigma) and according to the manufacturer’s instructions. cDNA was synthesized from total RNA using Improm-II^TM^ Reverse Transcriptase (Promega) with a random primer or using the ReverTra Ace® qPCR RT Kit (Toyobo). For quantitative real-time PCR, the experiment was performed as follows: quantitative real-time PCR was performed using THUNDERBIRD® SYBR® qPCR Mix (Toyobo) and according to the manufacturer’s instructions. All PCR products had a unique dissociation curve. Amplification was performed under the following conditions: 95 °C (10 min), followed by 40 cycles at 95 °C (30 s), then 58.5 °C (30 s). The threshold cycle (Ct) value for each gene was normalized to the Ct value for β-actin. Relative mRNA expression was calculated using the ΔΔCt method.

### Transwell migration and matrigel invasion assay

Migration assays were performed according to the manufacturer’s instructions. For transwell assay, siRNA transfected cells were incubated with control CM or Wnt3a-CM for 24 hr. Then, cells were trypsinized and counted. Cells (1 × 10^5^) in 0.3 ml of each conditioned media were seeded onto the upper chamber of cell culture inserts (SPL, 8 μm pore membrane Pore Size) and 0.7 ml of complete growth medium containing 10% FBS was added to the lower chamber. Following incubation for 24 h, non-migrated cells were removed from the upper chamber, and the migrated cells in the lower chamber were fixed with methanol, stained with 0.5% crystal violet and then photographed under a light microscope. For invasion assay, insert chambers were pre-coated with matrigel (BD Biosciences) diluted in serum-free medium. Remaining steps are the same as the protocol for transwell assay.

### Live confocal microscopy and confocal image analysis

All the confocal microscope images were acquired using a Leica TCS SP8 STED CW System and Leica DMI 6000 inverted fluorescent microscope (Leica). For observing and capturing green fluorescence of Alexa 488, the sample was excited via an Argon 488 nm laser with 10% of output and 20% of laser power settings. For red fluorescence of Alexa 568, the sample was excited using a DSPP 561 nm laser with 5% laser power setting. Photomultiplier (PMT) detector /Hybrid Detector setup (HyD, Hamamatsu) was used for detection of emission with the scan speed of 400 Hz using 8 × line average with 1 Airy unit pinhole setting. Emission spectral detection ranges were 500–550 nm for green fluorescence and 580–650 nm for red fluorescence. Nuclear co-localization of β-catenin and TFEB respectively labeled for green and red fluorescence were analyzed using MetaMorph software (Molecular Devices, USA). Intensity-based mean value of each fluorescent signal representing β-catenin and TFEB was automatically calculated with the equation of pixel sum intensity divided by a region of interest (ROI) area pixel. For molecular counting of β-catenin and TFEB areas, images were analyzed with IMARIS FL image analysis software V. 9.2 using the add spots and section mode. Estimated diameter of molecules for β-catenin and TFEB was determined in slice view as follows: the smallest XY dot in the images was ~250 nm and Z resolution of 600 nm. For live confocal microscope images, we used Zeiss AXIO Observer Z1. TFEB-EGFP stable cells were simultaneously imaged in a top-stage incubation system (Chamlide TCTM, LCI), which continuously provides 37 °C warmness and 5% CO_2_ gas incubation. IWR-endo or XAV939, Axin stabilizer were mixed with 2 ml Wnt3a-CM. The final concentration of IWR-endo was 10 μM and for XAV939, it was 2 μM.

### TCGA database analysis

For analyzing the correlation of expression levels between Axin2 and TFEB-mediated Wnt target genes or TFEB-mediated lysosomal gene, gene expression data sets from TCGA breast cancer, GDC TCGA LUSC and TCGA bladder cancer database were downloaded from the UCSC Xena browser (https://xena.ucsc.edu/). The correlation rates were analyzed using GraphPad Pearson correlation. For analyzing the Kaplan–Meier plot, survival data sets from TCGA breast cancer, GDC TCGA LUSC and TCGA bladder cancer database were downloaded from UCSC Xena browser (https://xena.ucsc.edu/).

## Supplementary information

Supplementaty Figure S1

Supplementaty Figure S2

Supplementaty Figure S3

Supplementaty Figure S4

Supplementaty Figure S5

Supplementaty Figure S6

Supplementaty Figure S7

Supplementaty Figure S8

Supplementary Tables

Supplementary Figure and Table Legends

## Data Availability

All datasets on which the conclusions of the paper rely are available to readers. The raw data for RNASeq analysis (shown in Fig. 4) can be accessible at https://www.ncbi.nlm.nih.gov/geo/query/acc.cgi?acc=GSE147769.
