# Supplementary figures and images for: PARsylated transcription factor EB (TFEB) regulates the expression of a subset of Wnt target genes by forming a complex with β-catenin-TCF/LEF1
Source: Cell Death Differ. 2021 Mar 22;28(9):2555–70. doi: 10.1038/s41418-021-00770-7 (PMC8408140; doi:10.1038/s41418-021-00770-7)

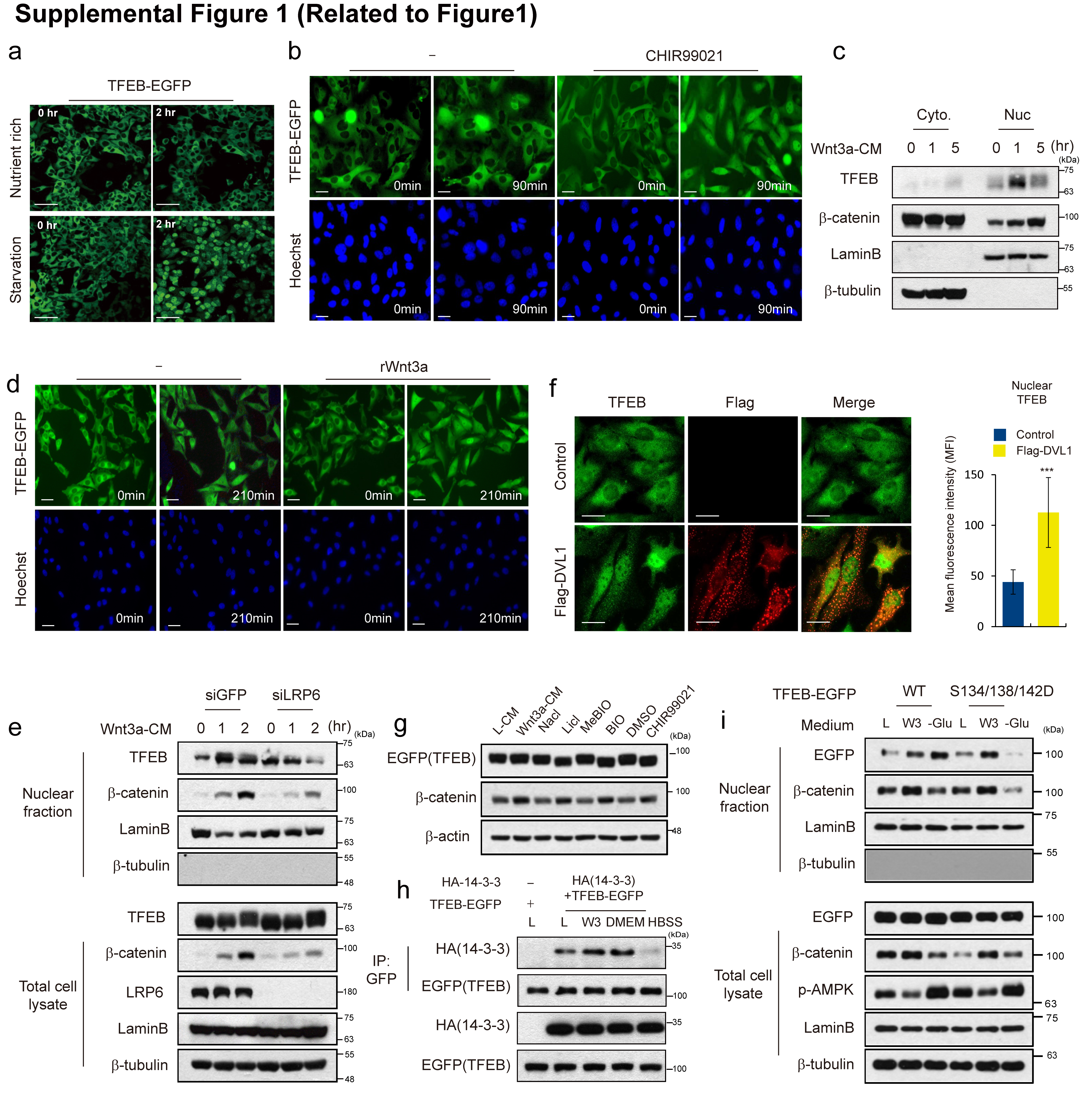

Supplement: Supplementary file 1 — Supplementaty Figure S1 [file 41418_2021_770_MOESM1_ESM.tif]

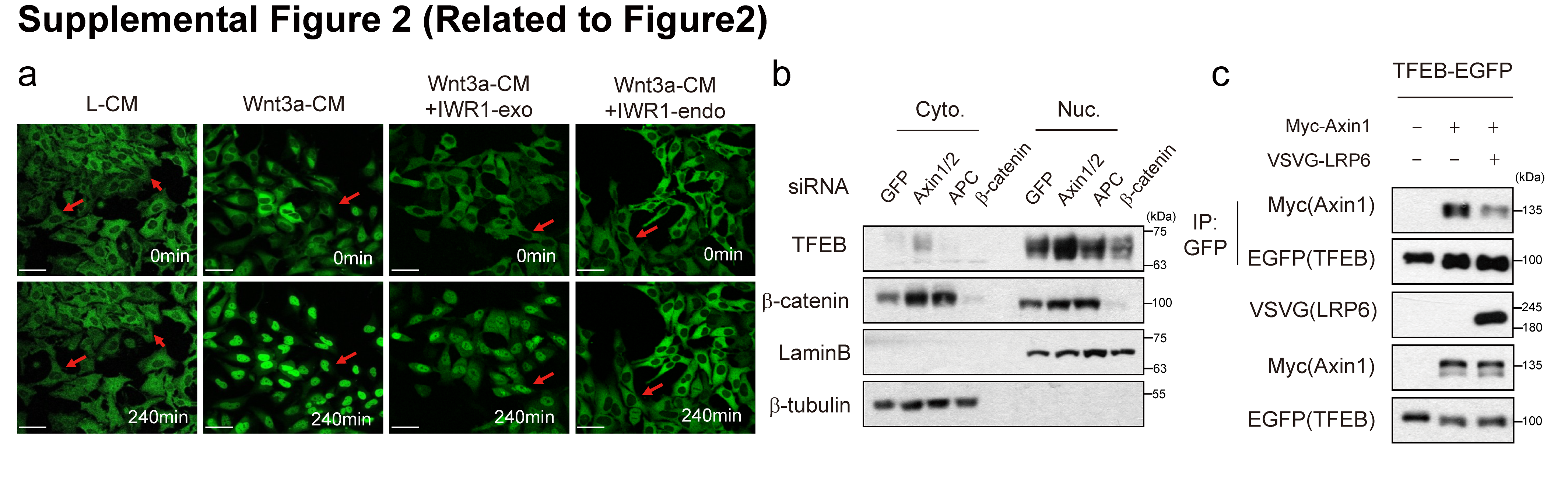

Supplement: Supplementary file 2 — Supplementaty Figure S2 [file 41418_2021_770_MOESM2_ESM.tif]

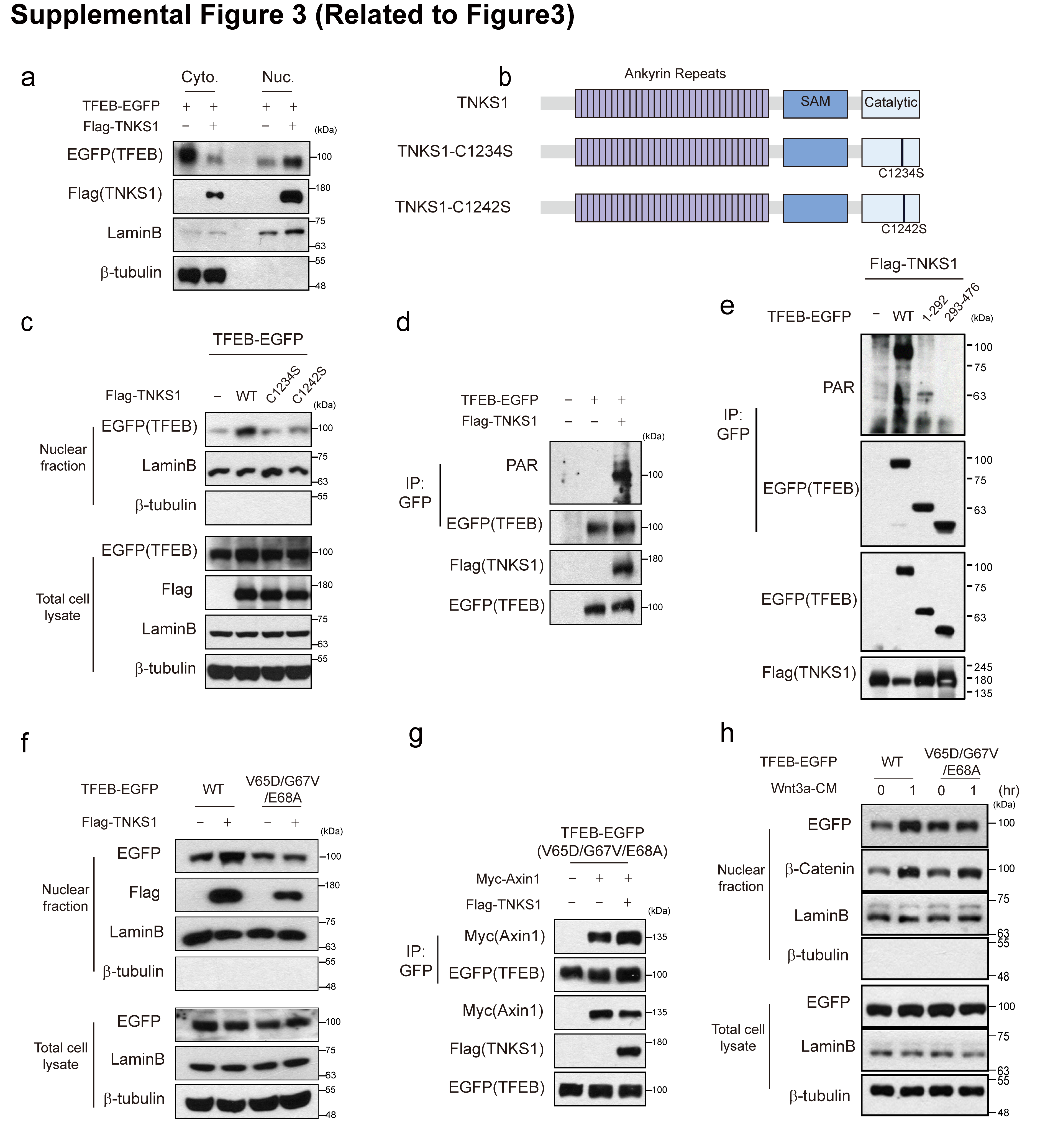

Supplement: Supplementary file 3 — Supplementaty Figure S3 [file 41418_2021_770_MOESM3_ESM.tif]

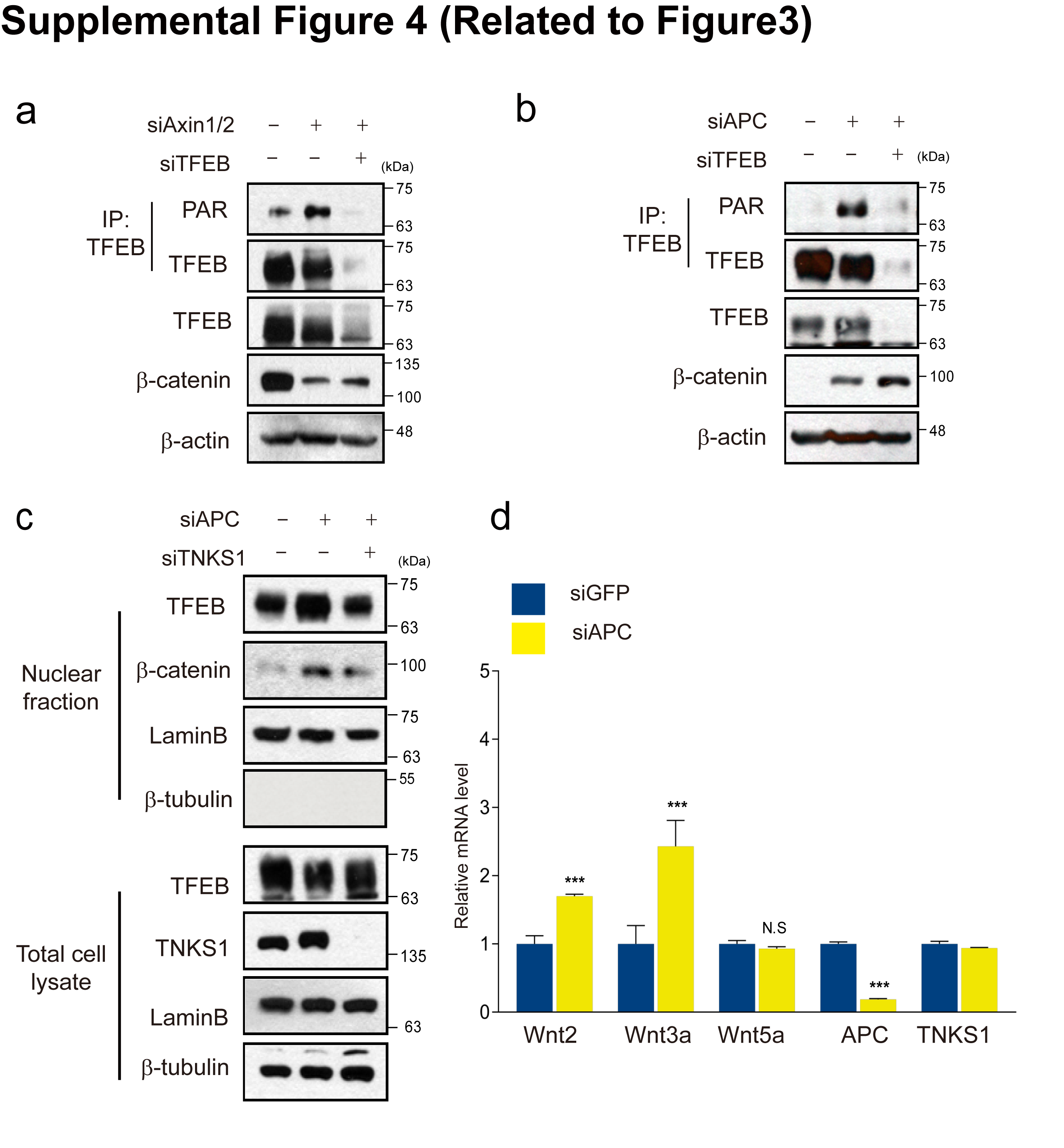

Supplement: Supplementary file 4 — Supplementaty Figure S4 [file 41418_2021_770_MOESM4_ESM.tif]

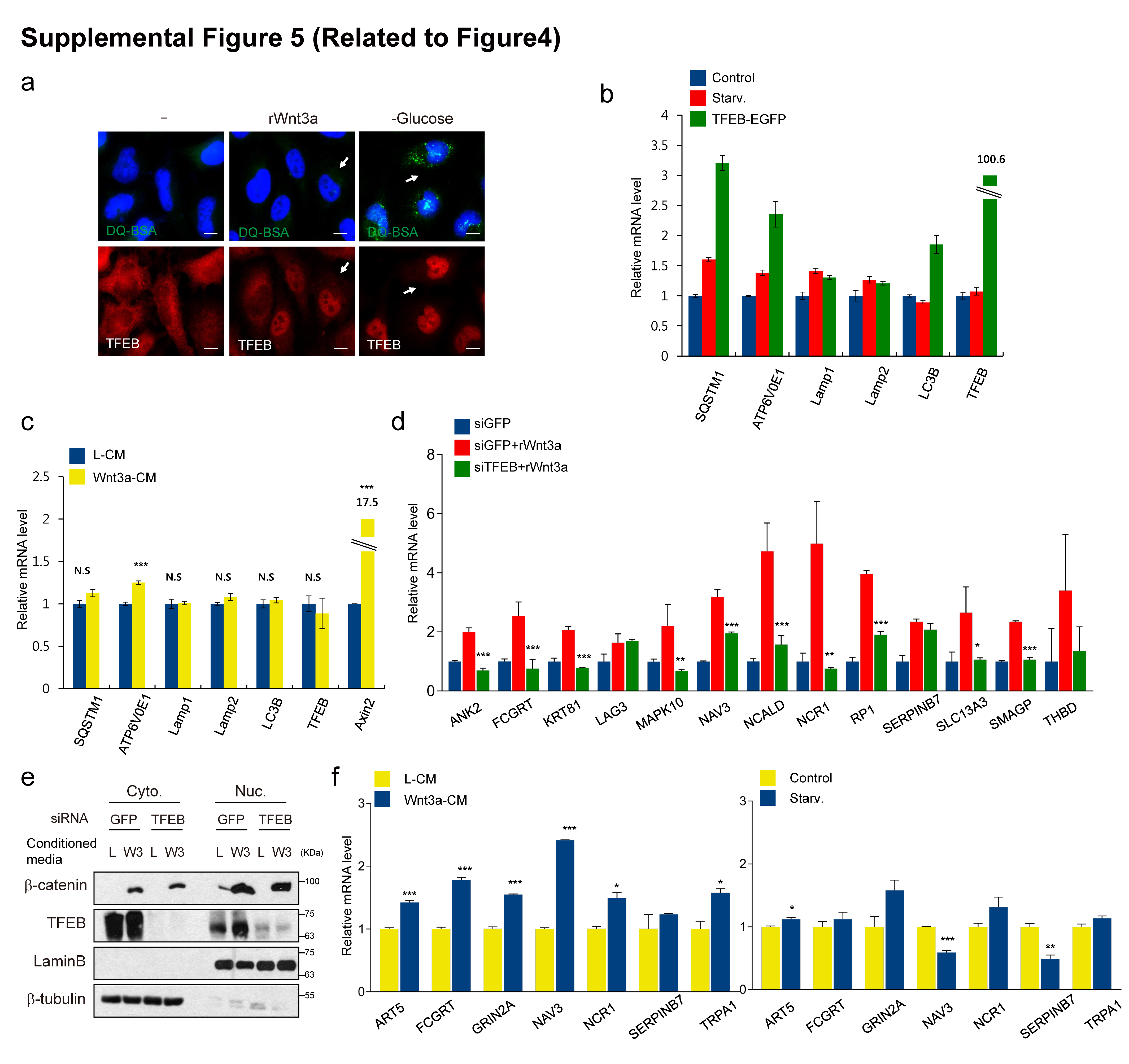

Supplement: Supplementary file 5 — Supplementaty Figure S5 [file 41418_2021_770_MOESM5_ESM.tif]

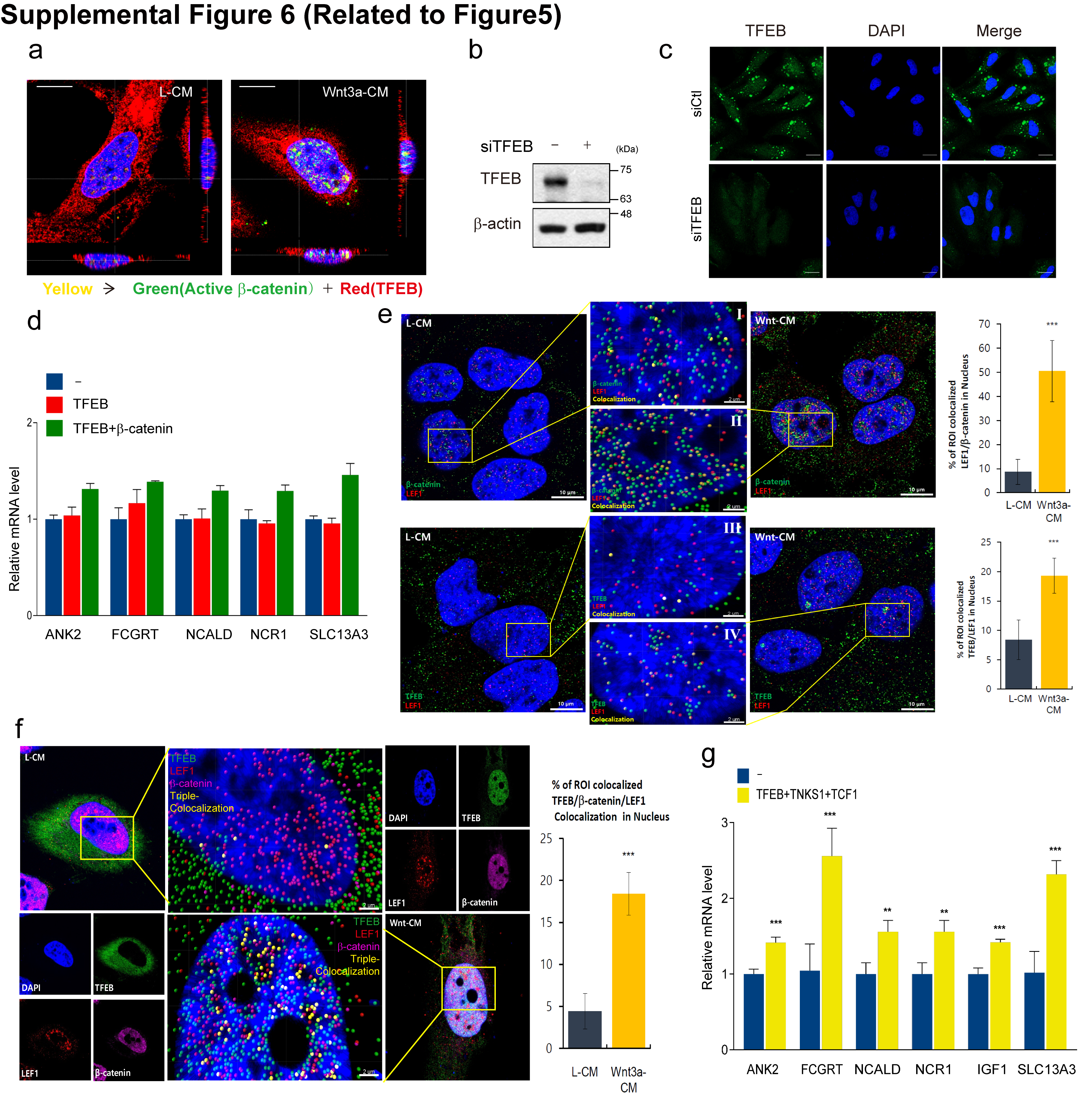

Supplement: Supplementary file 6 — Supplementaty Figure S6 [file 41418_2021_770_MOESM6_ESM.tif]

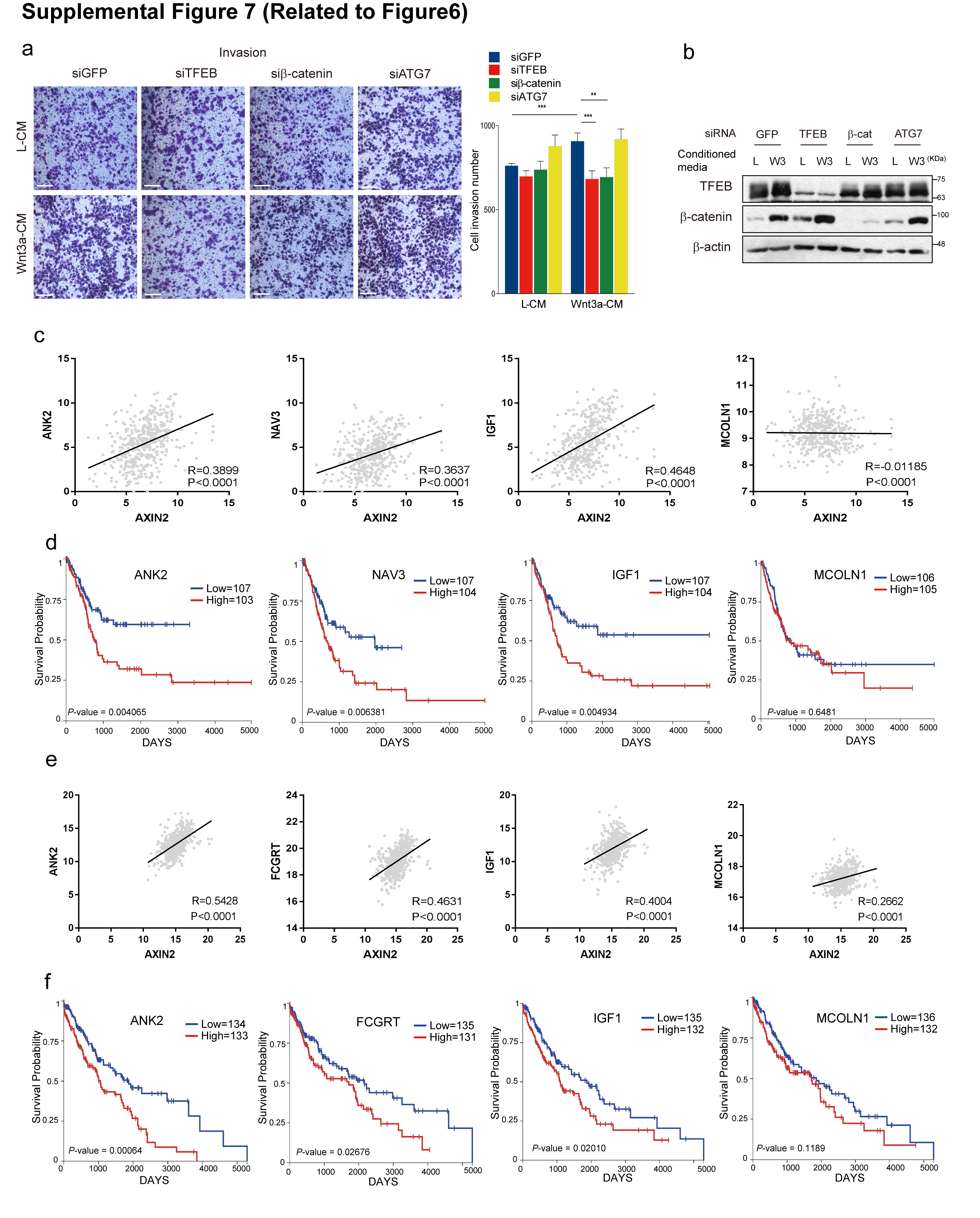

Supplement: Supplementary file 7 — Supplementaty Figure S7 [file 41418_2021_770_MOESM7_ESM.tif]

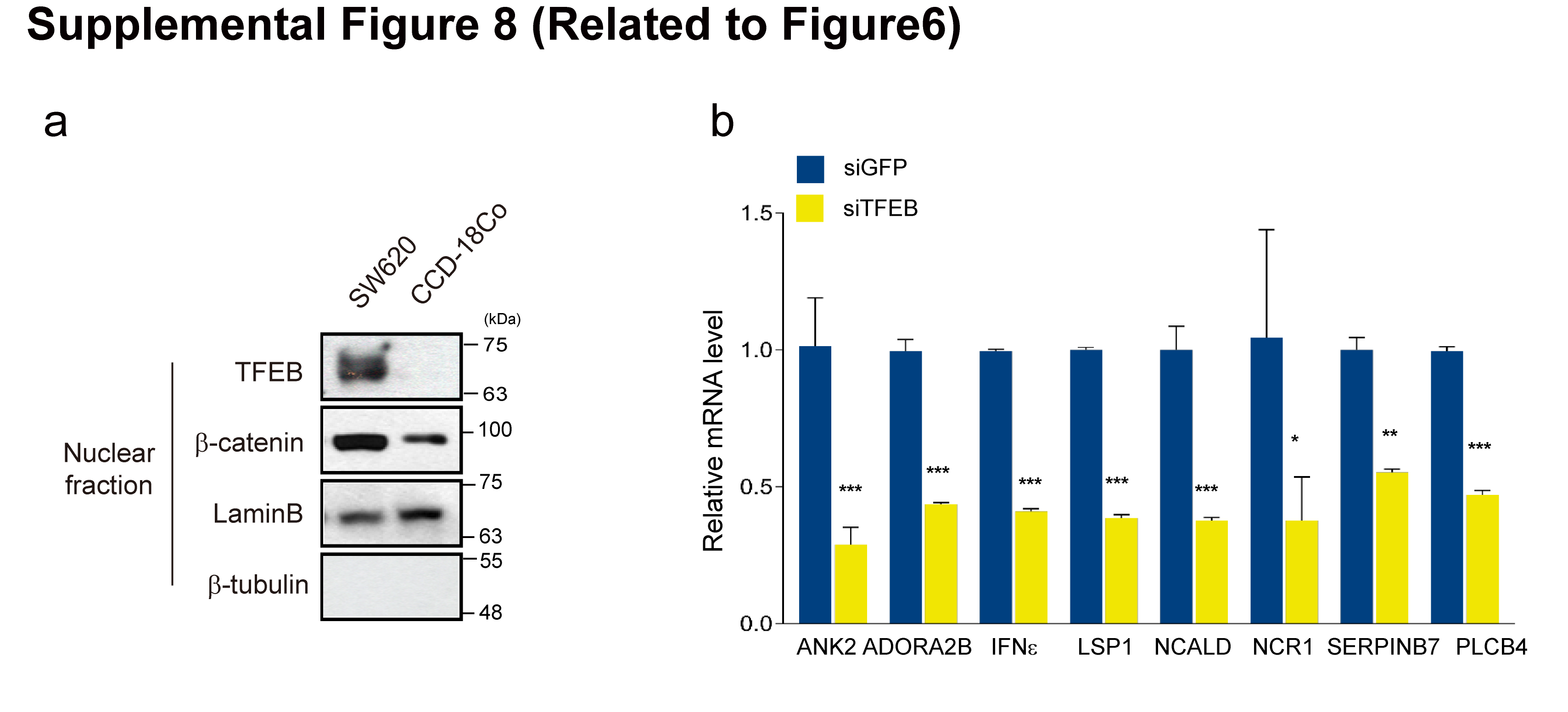

Supplement: Supplementary file 8 — Supplementaty Figure S8 [file 41418_2021_770_MOESM8_ESM.tif]
