# Supplementary material for: PARsylated transcription factor EB (TFEB) regulates the expression of a subset of Wnt target genes by forming a complex with β-catenin-TCF/LEF1
Source: Cell Death Differ. 2021 Mar 22;28(9):2555–70. doi: 10.1038/s41418-021-00770-7 (PMC8408140; doi:10.1038/s41418-021-00770-7)
Supplement: Supplementary file 9 — Supplementary Tables [file 41418_2021_770_MOESM9_ESM.docx]

**Supplemental Tables for**

**PARsylated transcription factor EB (TFEB) regulates the expression of a subset of Wnt target genes by forming a complex with β-catenin-TCF/LEF1**

Soyoung Kim^1^, Gahyeon Song^1^, Taebok Lee^2^, Minseong Kim^3^, Jeongrae Kim^4^, Hyeryun Kwon^1^, Jiyoung Kim^1^, Wonyoung Jeong^1^, Ukjin Lee^1^, Chaebin Na^1^, Sangwon Kang^5^, Wantae Kim^6^, Je Kyung Seong^7^, and Eek-hoon Jho^1^

^1^Department of Life Science, University of Seoul, 163 Seoulsiripdaero, Dongdaemun-gu, Seoul 02504, Republic of Korea

^2^Confocal Core Facility, Center for Medical Innovation, Seoul National University Hospital, Seoul 03082, Republic of Korea

^3^DKFZ-ZMBH Alliance, Deutsches Krebsforschungszentrum (DKFZ), Germany

^4^Department of Mathematics, University of Seoul, 163 Seoulsiripdaero, Dongdaemun-gu, Seoul 02504, Republic of Korea

^5^Research Center for Cell Homeostasis, Ewha Womans University, Seoul, 03760, Republic of Korea

^6^Department of Biochemistry, Chungnam National University, Daejeon 34134, Republic of Korea

^7^Laboratory of Developmental Biology and Genomics, College of Veterinary Medicine, Seoul National University, Seoul, 08826, Republic of Korea

Correspondence to

Eek-hoon Jho : [ej70@uos.ac.kr](mailto:ej70@uos.ac.kr), 82-2-6490-2671

**Supplemental Tables**

**Table S1. siRNA sequences used for knockdown, related to Material and Methods.**

| Name | Sequence |
| --- | --- |
| siRNA: human TFEB #1 | sense: 5’- AGACGAAGGUUCAACAUCA-3’  antisense: 5’- UGAUGUUGAACCUUCGUCU - 3’ |
| siRNA: human TFEB #2 | sense: 5’- GAACAAGUUUGCUGCCCACAU -3’  antisense: 5’- AUGUGGGCAGCAAACUUGUUC - 3’ |
| siRNA: human TFEB #3 | sense: 5’- CCGCCUGGAGAUGACCAACAA -3’  antisense: 5’- UUGUUGGUCAUCUCCAGGCGG - 3’ |
| siRNA: APC | sense: 5’- GAC GUU GCG AGA AGU UGG A -3’  antisense: 5’- UCC AAC UUC UCG CAA CGU C - 3’ |
| siRNA: β-catenin | sense: 5’- CCA AGA AGC AGA GAU GGC CCA GAA U -3’  antisense: 5’- AUU CUG GGC CAU CUC UGC UUC UUG G - 3’ |
| siRNA: Axin1 | sense: 5’-GGCAUAUCUGGAUACCUG -3’  antisense: 5’- GGAUACCUGCCGACCUUAA - 3’ |
| siRNA: Axin2 | sense: 5’- GAGUAGCCAAAGCGAUCTA -3’  antisense: 5’- CGAUCCUGUUAAUCCUUAU - 3’ |
| siRNA: TNKS1 | sense: 5’- CUA CAA CAG AGU UCG AAU A -3’  antisense: 5’- UAU UCG AAC UCU GUU GUA G - 3’ |
| siRNA: TNKS2 | sense: 5’- AGC UCA UAA UGA UGU UGU UGA AGU A -3’  antisense: 5’- UAC UUC AAC AAC AUC AUU AUG AGC U- 3’ |
| siRNA: TCF7 #1 | sense: 5’-GAAAUGCAUUCGGUACUUA -3’  antisense : 5’- UAA GUA CCG AAU GCA UUU C - 3’ |
| siRNA: TCF7 #2 | sense: 5’- CUACGAACAUUUCAGCAGU -3’  antisense: 5’- ACU GCU GAA AUG UUC GUA G - 3’ |
| siRNA: LRP6 | sense: 5’- ACAUUGUUCUGCAGUUAGA -3’  antisense: 5’- UCUAACUGCAGAACAAUGU - 3’ |
| siRNA: siATG7 | sense: 5’- AAG GAG UCA CAG CUC UUC CUU -3’  antisense: 5’- AAG GAA GAG CUG UGA CUC CUU- 3’ |

**Table S2. Antibodies for western blot and immunoprecipitation, related to Material and Methods.**

| **Antibodies** | | |
| --- | --- | --- |
| **Resources** | **Manufacturer** | **Catalog** |
| Anti-HA-antibody | Santa Cruz | Cat#sc-7392 |
| Lamin B antibody | Santa Cruz | Cat#sc-6217 |
| TNKS1 antibody | Santa Cruz | Cat#sc-365897 |
| EGFP antibody | Santa Cruz | Cat#sc-9996 |
| EGFP antibody | Invitrogen | Cat#A11122 |
| TFEB antibody | Cell Signaling | Cat#4240s |
| TFEB antibody | Bethyl Laboratories | Cat#A303-673A |
| Axin1 antibody | Cell signaling | Cat#2087s |
| β-actin antibody | Sigma | A5441 |
| Flag antibody | Sigma | Cat#F3165 |
| VSVG antibody | Sigma | Cat#v4888 |
| Poly(ADP-Ribose)Polymer antibody | Abcam | Cat#Ab14459 |
| Myc antibody | Abm | Cat#G019 |
| β-catenin antibody | BD Bioscience | Cat#610154 |
| active-β-catenin antibody | Millipore | 05-665 |
| β-tubulin antibody | Gene Tex | Cat#GTX101279 |
| IgG antibody | Stratagene |  |

**Table S3. Primers used for real time PCR, related to Material and Methods.**

| Name | Sequence |
| --- | --- |
| ADORA2B | F : GGG GTG GAA CAG TAA AGA CAG  R: CAG CAG CTT TCA TTC GTG GTT |
| ART5 | F : AAGCGTCGAGGGCTTACCT  R : GGTGTTCGATGAGTTGGTGTAGA |
| ANK2 | F :ACTGAGAGTGGTTTTACCCCT  R : CAGCTCCCCGGTTTAGAAGA |
| ATG7 | F : CAGTTTGCCCCTTTTAGTAGTGC  R : CCAGCCGATACTCGTTCAGC |
| CCDC146 | F : GGCAGCGTTAAAAGCCAAGTA  R : TAGCAGTTGGACCTCTGACTC |
| FCGRT | F : AAG GCC ACG GGG ATA CAG  R : CGG AGT CCA CAA CTT GGA G |
| GRIN2A | F : TCA TGC AGG ATT ATG ACT GGC A  R : TGT GGT CTT GAC GAA GCT GAT |
| KRT81 | F : AGAGCAACAGCTGAGAACGAG  R : GTCTGACTTGCGGAGGTAGG |
| IGF1 | F : TGTGGAGACAGGGGCTTTTA  R : ATCCACGATGCCTGTCTGA |
| ITGA10 | F : ACT TAG GTG ACT ACC AAC TGG G  R : CCA CAA GCA CGA GAC CAG A |
| LSP1 | F : GGG TGA GGT ACA GGC TCA GT  R : CTG CTC CCA GAG GCT TTT C |
| Wnt2 | F : ATGTCACCCGGATGACCAAG  R : TCCAGAGCTTCCAGGCAGTC |
| Wnt3a | F : TGTTGGGCCACAGTATTCCT  R : GGGCATGATCTCCACGTAGT |
| Wnt5a | F : GCCCAGGTTGTAATTGAAGC  R : TGGCACAGTTTCTTCTGTCC |
| NAV3 | F : TTATCTCGCTACAAGCAGCAAC  R : GGGGAAGCGTGAGTAACTCG |
| NCR1 | F : TGG ACC CGA AGT GAT CTC G  R : TCC TTG AGC AGT AAG AAC ATG C |
| RUNDC3B | F : TCGGTCTTGCTTTGAGACAA  R : CTGCAACTTTGGGAAATCTCTT |
| RP1 | F : ATG AGT GAT ACC CCT TCT ACT GG  R : ACA GGA TGA GTG AGG CTC AAA T |
| SERPINB7 | F : TAAGCTCATCTGCTGTAATGGTG  R : GGCAATTTATGGTTTCGCTCTTG |
| SLC13A3 | F : TTGGCTATGAATACCTGGGCA  R : GGGTGGCAATGCTGTGACA |
| SMAGP | F : ACCAGCCTCCTGACTACTCC  R : GGGGTGGTCATCAGTTCTTCT |
| TFEB | F : CCT GGA GAT GAC CAA CAA GCA G  R: TGT GAT TGT CTT TCT TCT GCC G |
| THBD | F : ACC TTC CTC AAT GCC AGT CAG  R : CGT CGC CGT TCA GTA GCA A |
| TNKS | F : TGGTGCTGATGTTCATGCAAA  R : ACAAGCTCCATGCTTTAGTAGC |
| PLCB4 | F : TATTCGGTCGGGAGCCATAC  R : GACACAAACTATCCGCCCTTC |
| β-actin | F : GCG GGA AAT CGT GCG TGA CAT T  R: GAT GGA GTT GAA GGT AGT TTC GTG |
