# Supplementary material for: PARsylated transcription factor EB (TFEB) regulates the expression of a subset of Wnt target genes by forming a complex with β-catenin-TCF/LEF1
Source: Cell Death Differ. 2021 Mar 22;28(9):2555–70. doi: 10.1038/s41418-021-00770-7 (PMC8408140; doi:10.1038/s41418-021-00770-7)
Supplement: Supplementary file 10 — Supplementary Figure and Table Legends [file 41418_2021_770_MOESM10_ESM.docx]

**Supplemental Figure and Table legend for**

**PARsylated transcription factor EB (TFEB) regulates the expression of a subset of Wnt target genes by forming a complex with β-catenin-TCF/LEF1**

Soyoung Kim^1^, Gahyeon Song^1^, Taebok Lee^2^, Minseong Kim^3^, Jeongrae Kim^4^, Hyeryun Kwon^1^, Jiyoung Kim^1^, Wonyoung Jeong^1^, Ukjin Lee^1^, Chaebin Na^1^, Sangwon Kang^5^, Wantae Kim^6^, Je Kyung Seong^7^, and Eek-hoon Jho^1^

^1^Department of Life Science, University of Seoul, 163 Seoulsiripdaero, Dongdaemun-gu, Seoul 02504, Republic of Korea

^2^Confocal Core Facility, Center for Medical Innovation, Seoul National University Hospital, Seoul 03082, Republic of Korea

^3^DKFZ-ZMBH Alliance, Deutsches Krebsforschungszentrum (DKFZ), Germany

^4^Department of Mathematics, University of Seoul, 163 Seoulsiripdaero, Dongdaemun-gu, Seoul 02504, Republic of Korea

^5^Research Center for Cell Homeostasis, Ewha Womans University, Seoul, 03760, Republic of Korea

^6^Department of Biochemistry, Chungnam National University, Daejeon 34134, Republic of Korea

^7^Laboratory of Developmental Biology and Genomics, College of Veterinary Medicine, Seoul National University, Seoul, 08826, Republic of Korea

Correspondence to

Eek-hoon Jho : [ej70@uos.ac.kr](mailto:ej70@uos.ac.kr), 82-2-6490-2671

**Supplementary Figure Legends**

**Figure S1. related to Figure 1.**

**(a)** Nutrient deprivation induces nuclear localization of TFEB-EGFP. TFEB-EGFP stably transfected HeLa cells were treated with DMEM or HBSS starvation media. Cell fluorescence live images were captured using a confocal microscope. Scale bar, 100μm. **(b)** Treatment of GSK3β inhibitor induced nuclear localization of TFEB-EGFP. Live cell images of TFEB-EGFP in TFEB-EGFP stably transfected HeLa cells upon treatment with DMSO or CHIR99021 (2 μM). Scale bar, 20μm. **(c)** Treatment of Wnt3a-CM induced nuclear localization of endogenous TFEB. Cytosolic and nuclear fraction of HeLa cell lysates were used for immunoblotting with the indicated antibodies. β-tubulin and Lamin B were used as markers for cytosolic and nuclear fractions, respectively. **(d)** Treatment with recombinant human Wnt3a (rWnt3a) induced nuclear localization of TFEB-EGFP. TFEB-EGFP stably transfected HeLa cells were exposed to rWnt3a (100 ng/ml). Nuclei were stained by Hoechst. Scale bar, 50μm. **(e)** Knockdown of LRP6, a Wnt co-receptor, blocked the increase in nuclear TFEB levels induced by treatment with Wnt3a-CM. LRP6 was knockdown in HeLa cells and treated with Wnt3a-CM at respective time as indicated in the figure. Cytosolic and nuclear fraction of HeLa cell lysates were used for immunoblotting with the indicated antibodies. **(f)** Overexpression of DVL induced nuclear localization of endogenous TFEB. Immunofluorescence analysis was performed in HeLa cells transfected with Flag-DVL1. Quantification of nuclear TFEB was shown in right panel. Data are presented as mean ± SEM. ***P<0.005 (Student’s t-test). Scale bar, 20μm. **(g)** Mobility shift of TFEB was induced by GSK3β inhibitor treatment but not with Wnt3a-CM. TFEB-EGFP stably transfected HeLa cells were treated with for 3 hours with DMSO or GSK3β inhibitors including CHIR99021 (2 μM) or BIO (2 μM) or LiCl (20 mM). **(h)** Treatment with Wnt3a-CM did not affect the interaction between TFEB and 14-3-3. HEK293T cells were transfected with TFEB-EGFP and HA-14-3-3 or control vector. After 16 hours, the cells were treated with Wnt3a-CM or HBSS. Lysates were subjected to immunoprecipitation (IP) with the anti-GFP antibody, followed by immunoblotting with the indicated antibody. **(i)** Wnt3a-CM treatment enhanced the nuclear levels of a phosphomimetic mutant TFEB-EGFP form, S134/138/142D, while glucose starvation had no effect. HeLa cells transfected with TFEB-EGFP or TFEB-EGFP(S134/138/142D) were cultured in Wnt3a-CM or glucose deprivation medium for 4 hours. Cytosolic and nuclear fraction of cell lysates were used for immunoblotting with the indicated antibodies.

**Figure S2. related to Figure 2.**

**(a)** Treatment of cells with IWR-1 endo or XAV939 blocked Wnt3a-CM mediated nuclear localization of TFEB. TFEB-EGFP stably transfected HeLa cells were treated with L-CM or Wnt3a-CM or Wnt3a-CM+IWR-exo (10 nM) or Wnt3a+IWR endo (10 nM). Scale bar, 50μm. **(b)** Knockdown of Axin1/2 or APC but not β-catenin induced nuclear localization of TFEB. Nuclear and cytosolic fractions of lysates from HeLa cells were used and immunoblotted with antibodies indicated in the figure. **(c)** The interaction between Axin and TFEB was reduced by ectopic expression of VSVG-LRP6. TFEB-EGFP and Myc-Axin1-expressing HEK293T cells were co-transfected with VSVG-LRP6. The cell lysates were immunoprecipitated with the anti-GFP antibody and followed by immunoblotting with the indicated antibodies.

**Figure S3. related to Figure 3.**

**(a)** Co-overexpression with Flag-TNKS1 and TFEB-EGFP increased the levels of nuclear TFEB-EGFP. TFEB-EGFP and Flag-TNKS1 were co-transfected into HEK293T cells. Nuclear and cytosolic fractions of lysates were immunoblotted with the antibodies indicated in the Figure. **(b)** Schematic diagram of wild type and mutants of TNKS1 (SAM, sterile α motif; C, Cys; S, Ser). **(c)** Overexpression of wild type TNKS-1, but not PARP activity deficient TNKS1 mutant, increased the levels of nuclear TFEB. TFEB-EGFP with Flag-TNKS1 or TNKS1 mutants was co-transfected into HEK293T cells. Nuclear fractions and total lysates were immunoblotted with the antibodies indicated in the Figure. **(d)** Overexpression of TNKS1 induced PARsylation of TFEB. TFEB-EGFP and Flag-TNKS1 were co-transfected into HEK293T cells. Cells were lysed with buffer containing poly(ADP-ribose) glycohydrolase inhibitor, ADP-HPD (5 μM). Cell lysate were immunoprecipitated with anti-GFP antibody and immunoblotted with the PARsylation (PAR) antibody. **(e)** TNKS1 PARsylates N-terminal domain of TFEB. Flag-TNKS1 and TFEB-EGFP or deletion constructs were co-transfected into HEK293T cells and PARsylation assay was performed as described in **(d)**. **(f)** Overexpression of TNKS1 increased the levels of nuclear TFEB, but not TNKS-binding deficient TFEB mutant (TFEB DVA). Flag-TNKS1 with TFEB-EGFP or TFEB-EGFP mutant was co-transfected into HEK293T cells. Nuclear fractions and total lysates were immunoblotted with the antibodies indicated in the Figure. **(g)** TFEB DVA did not dissociate from Axin under TNKS1 overexpression setting. Plasmids indicated in the Figure were transfected into HEK293T cells. Cell lysates were immunoprecipitated with anti-GFP antibody and immunoblotted with the indicated antibodies. **(h)** Treatment of Wnt3a-CM increased the levels of nuclear TFEB, but not TNKS-binding deficient TFEB mutant (TFEB DVA). Flag-TNKS1 with TFEB-EGFP or TFEB-EGFP mutant was co-transfected into HEK293T cells. Nuclear fractions and total lysates were immunoblotted with the antibodies indicated in the Figure.

**Figure S4. related to Figure 3.**

**(a-b)** Knockdown of Axin1/2 or APC increased the levels of PARsylation on endogenous TFEB. Axin1/2 **(a)** or APC **(b)** were knockdown in HeLa cells. Cell lysates were immunoprecipitated with TFEB antibody and immunoblotted with indicated antibodies. To show specificity of PARsylated signal TFEB was knockdown. **(c)** Knockdown of TNKS1 reduced APC knockdown-mediated induction of the nuclear TFEB level. APC and TNKS1 were knockdown in HeLa cells. Nuclear fractions and total lysates were immunoblotted with the antibodies indicated in the figure. **(d)** Knockdown of APC enhanced the expression of Wnt2 and Wnt3a. APC was knockdown in HeLa cells and Real time PCR analysis was performed. Data are presented as mean ± SEM. ***P<0.005 (Student’s t-test).

**Figure S5. related to Figure 4.**

**(a)** Wnt3a-mediated nuclear-localized TFEB does not induce lysosomal biogenesis. HeLa cells were treated with rWnt3a in cultured media or glucose free media. Functional lysosomes were visualized by incubation with the indicated media including BSA-DQ. Endogenous TFEB were labeled with Alexa546-Red. Both the treatment of rWnt3a and glucose starvation induced nuclear localization of endogenous TFEB (Bottom). Glucose starvation, but not Wnt3a-CM, induced active lysosome formation (Top). Scale bar, 10μm. **(b)** Level of mRNAs encoding lysosomal genes was increased by TFEB overexpression and glucose deprivation. HeLa cells were cultured with control or glucose free media for 4 hours or transfected with TFEB-EGFP. Expression of well-known TFEB-dependent lysosomal target genes was measured by Real time PCR analysis. **(c)** Level of mRNAs encoding lysosomal genes was not increased upon treatment with Wnt3a-CM. HeLa cells were treated with L-CM or Wnt3a-CM for 4 hours and qPCR was performed to measure expression of lysosomal genes. **(d)** siGFP or siTFEB transfected cells were treated with rWnt3a. HeLa cells transfected with siGFP or siTFEB were treated with rWnt3a for 8 hours. qPCR was performed to measure expression of TFEB-dependent lysosomal target genes. **(e)** Knockdown of TFEB did not affect the subcellular localization of β-catenin. HeLa cells transfected with siGFP or siTFEB were treated with L-CM or Wnt3a-CM for 4 hours. Nuclear and cytosolic fractions of lysates were immunoblotted with the antibodies indicated in the Figure. **(f)** Only treatment of Wnt3a-CM, but not starvation media, induced the expression of “TFEB-mediated Wnt target genes”. HeLa cells were cultured in Wnt3a-CM or glucose free media for 6 hours. qPCR was performed to measure expression of TFEB-dependent lysosomal target genes.

**Figure S6. Related to Figure 5.**

**(a**) Endogenous TFEB co-localized with active β-catenin in the nucleus. HeLa cells were treated with L-CM or Wnt3a-CM, respectively, for 4 hours. Confocal z-stack images were captured using Zeiss LSM 510 Meta for 3D image visualization. Active β-catenin and TFEB were labeled with Alexa 488-Green and Alexa 568-Red antibodies, respectively. Yellow-labeled area depict co-localization between active β-catenin and TFEB. Scale bar, 10μm. **(b**) Knockdown of TFEB significantly reduces its levels indicating the specificity of TFEB antibody used here. **(c)** Knockdown of TFEB reduced the perinuclear positive signals detected by Alexa 488 as a secondary antibody. **(d)** Overexpression of TFEB and β-catenin did not induce expression of “TFEB-mediated Wnt target genes”. qPCR was performed for TFEB-mediated Wnt target genes in TFEB-EGFP expressing HeLa cells co-transfected with HA-β-catenin. **(e)** Wnt3a-CM treatment enhanced the interactions between β-catenin and LEF1, and between LEF1 and TFEB. Colocalization spot analysis. β-catenin and LEF1; TFEB and LEF1 levels in HeLa cells were detected with Alexa 488 and Alexa 568, respectively. Estimated XY diameter of each sphere (I, II, III and IV) is 0.25 µm and z diameter is 0.5 µm, and PSF was elongated along the z-axis using Imaris 9.2 image analysis software (Oxford Instruments, UK). Colocalized spots are shown in yellow. Quantification of colocalization between LEF1 and β-catenin; TFEB and LEF1 in nuclear region is shown in right panel. **(f)** Wnt3a-CM treatment enhanced the triple-colocalization of β-catenin/LEF1/TFEB in the nuclei. Triple-colocalization of TFEB (green), LEF1 (red) and β-catenin (magenta) in HeLa cells. Fluorescently labeled spots were converted to spheres with estimated XY (0.25 µm) and Z (0.5 µm) in diameters using Imaris 9.2 image analysis software (Oxford Instruments, UK). Triple-colocalizated spots are shown in yellow. Quantification of triple-colocalization among TFEB, LEF1 and β-catenin in nucleus is shown in right panel. **(g)** Co-expression of TFEB, TNKS1, and TCF1 enhanced the expression of “TFEB-mediated Wnt target genes”. qPCR was performed for TFEB-mediated Wnt target genes in HeLa cells co-transfected with TFEB-EGFP, HA-TNKS1 and FLAG-TCF1. Data are presented as mean ± SEM. **P<0.01 and ***P<0.005 (Student’s t-test).

**Figure S7. Related to Figure 6**

**(a)** siRNA mediated knockdown of TFEB or β-catenin but not Atg7 impaired cancer cell matrigel invasion induced by Wnt3a-CM in HeLa cells. Quantification of number of invaded cells from three randomly selected regions are shown in the graph. Scale bar, 100μm. **(b)** Western blot analysis revealed that the protein expression of TFEB or β-catenin was reduced after the transfection with each siRNA. **(c)** Analysis of TCGA gene expression data set of bladder cancer patients show a positive correlation between Axin2 and TFEB-mediated Wnt target gene (ANK2, NAV3, IGF1) but not TFEB-mediated lysosomal gene (MCOLN1). **(d)** Kaplan-Meier plot shows that the high expression of TFEB-mediated Wnt target genes but not lysosomal gene is associated with poor prognosis of bladder cancer patients. **(e)** Analysis of GDC TCGA gene expression data set of lung squamous carcinoma (LUSC) patients show a positive correlation between Axin2 and TFEB-mediated Wnt target genes (ANK2, FCGRT, IGF1) but not lysosomal gene (MCOLN1). **(f)** Kaplan-Meier plot shows that high expression of TFEB-mediated Wnt target genes but not lysosomal gene is associated with poor prognosis of LUSC patients. The Correlation coefficients (*r*) in this figure were calculated by Pearman’s linear correlation. Two-tailed *P*-value was used for analyzing statistical significance.

Data information: In **(a)**, data are presented as mean ± SEM. *P<0.05, **P<0.01 and ***P<0.005 (Student’s t-test).

**Figure S8. Related to Figure 6**

**(a)** The nuclear TFEB level in SW620 (colon cancer cell line) cells was much higher than in CCD-18Co (normal colon cell line) cells. **(b)** Knockdown of TFEB in HT29 cells (a colorectal cancer cell line with an APC mutation) resulted in reduced expression of ‘TFEB mediated Wnt target genes’. qPCR for the genes depicted in the graph was performed upon transfection of siGFP or siTFEB RNA in HT29 cells.

**Supplementary Table Legends**

**Table S1. siRNA sequences used for knockdown, related to Material and Methods.**

Table S1 shows the siRNA sequences used in this study.

**Table S2. Antibodies for western blot and immunoprecipitation, related to Material and Methods.**

Table S2 shows the antibodies list used in this study.

**Table S3. Primers used for real time PCR, related to Material and Methods.**

Table S3 shows the primers for real time PCR used in this study.
